# A systematic review of animal predation creating pierced shells: implications for the archaeological record of the Old World

**DOI:** 10.7717/peerj.2903

**Published:** 2017-01-17

**Authors:** Anna Maria Kubicka, Zuzanna M. Rosin, Piotr Tryjanowski, Emma Nelson

**Affiliations:** 1 Independent researcher, Poznań, Poland; 2 Department of Cell Biology, Adam Mickiewicz University in Poznań, Poznań, Poland; 3 Institute of Zoology, Poznań University of Life Sciences, Poznań, Poland; 4 School of Medicine, University of Liverpool, Liverpool, United Kingdom; 5 Department of Archeology, Classics and Egyptology, University of Liverpool, Liverpool, United Kingdom

**Keywords:** Scaphopoda, Gastropoda, Bivalvia, Shell beads, Interspecies interactions, Jewellery, Predators

## Abstract

**Background:**

The shells of molluscs survive well in many sedimentary contexts and yield information about the diet of prehistoric humans. They also yield evidence of symbolic behaviours through their use as beads for body adornments. Researchers often analyse the location of perforations in shells to make judgements about their use as symbolic objects (e.g., beads), the assumption being that holes attributable to deliberate human behaviour are more likely to exhibit low variability in their anatomical locations, while holes attributable to natural processes yield more random perforations. However, there are non-anthropogenic factors that can cause perforations in shells and these may not be random. The aim of the study is compare the variation in holes in shells from archaeological sites from the Old World with the variation of holes in shells pierced by mollusc predators.

**Methods:**

Three hundred and sixteen scientific papers were retrieved from online databases by using keywords, (e.g., ‘shell beads’; ‘pierced shells’; ‘drilling predators’); 79 of these publications enabled us to conduct a systematic review to qualitatively assess the location of the holes in the shells described in the published articles. In turn, 54 publications were used to assess the location of the holes in the shells made by non-human predators.

**Results:**

Almost all archaeological sites described shells with holes in a variety of anatomical locations. High variation of hole-placement was found within the same species from the same site, as well as among sites. These results contrast with research on predatory molluscs, which tend to be more specific in where they attacked their prey. Gastropod and bivalve predators choose similar hole locations to humans.

**Discussion:**

Based on figures in the analysed articles, variation in hole-location on pierced shells from archaeological sites was similar to variation in the placement of holes created by non-human animals. Importantly, we found that some predators choose similar hole locations to humans. We discuss these findings and identify factors researchers might want to consider when interpreting shells recovered from archaeological contexts.

## Introduction

The adornments of prehistoric people play an important role in our understanding of the evolution of human behaviour (Bednarik, 2001; Szabo, Brumm & Bellwood, 2007; Gutiérrez-Zugasti & Cuenca-Solana, 2013) because they can indicate evolutionary changes in the ethno-linguistic diversity of early humans (Vanhaeren & D’Errico, 2006; Schick & Toth, 2013; Stiner, 2014). These findings help anthropologists to construct a picture of the life of prehistoric human groups, and can give insights into their social status (Bednarik, 1998; Stiner, 1999; Vanhaeren & D’Errico, 2005), group membership, age or marital status (Kuhn et al., 2001). Molluscs are among the most robust material remains. Shells fashioned into personal adornments survive well in most sedimentary contexts (Bar-Yosef-Mayer & Beyin, 2009; Lombardo et al., 2013) and can be interpreted in various ways, depending on the context of the find. Usually the deposits are associated with graves (Vanhaeren et al., 2004; Vanhaeren & D’Errico, 2005), human shelters (Kuhn et al., 2001) and hearths (Douka et al., 2014). Some of the earliest forms of body adornment are shell beads that date back to ∼75 Kya (Henshilwood et al., 2004) and ∼82 Kya (Bouzouggar et al., 2007), possibly even 100–130 Kya (Vanhaeren et al., 2006) or earlier (Bednarik, 2015). However, some researchers argue that this “modern behaviour” was probably established earlier than is reflected in the archaeological record, and is simply not visible due to taphonomic processes (Bowdler & Mellars, 1990; Noble & Davidson, 1996; Botha, 2008; Botha, 2010). Teasing apart pre-depositional effects in mollusc remains, however, can be made more difficult because predators can produce modifications which are similar to those produced by humans through their ability to make holes in shells.

Researchers use detailed analyses of adornments, radiometric dates and stratigraphic information to explain innovations in shell beads and the spread of cultural traditions (Kuhn et al., 2001). The location of piercings in shells can provide information on the placement of the shell bead within the finished adornment (e.g., a necklace; Baysal, 2013; Stiner, Kuhn & Güleç, 2013). Indications of human manipulation can also be detected, such as striations indicating rotary drilling by a tool (Zilhão et al., 2010), notches close to the perforation that might indicate the presence of a suspension system (e.g., cord) and the direction the traction was exerted (Cristiani, 2012). Researchers also use experiments to understand shell anatomy (e.g., mineralogy and structure) and the processes involved in the production of piercings (e.g., Beyin, 2010; Nigra & Arnold, 2013; Tátá et al., 2014; Joordens et al., 2015). Microscopy can provide evidence of the shape of the tools used for piercing shells, as well as other tell-tale signs of human activity (D’Errico et al., 2005; Nigra & Arnold, 2013). For example, piercings are often examined for the presence of residues, such as ochre or polishing by the cord (D’Errico et al., 2005; D’Errico et al., 2009; Stiner, Kuhn & Güleç, 2013). Similarly, microscopic analyses of naturally made holes in molluscs provide insight (e.g., Li, Young & Zhan, 2011; Gorzelak et al., 2013).

Based upon this kind of painstaking evidence-gathering, experts make judgements as to whether perforations in shells from archaeological sites are anthropogenic in origin or formed by natural processes (D’Errico et al., 2005), such as those made by hole-boring predators or parasites (Kowalewski, 2004; Li, Young & Zhan, 2011) or taphonomic processes (e.g., water erosion, crushing, diagenesis; Peacock et al., 2012; Gorzelak et al., 2013). While the location and type of the perforation is only part of a raft of evidence that indicates an operational chain (starting with the collection of the raw material, followed by the manufacture and use, and ending with its discard), some researchers have proposed that the anatomical locations of holes pierced in shells by humans exhibit low variability whereas holes made by non-human animals yield more random perforations (Stiner, 1999; Bouzouggar et al., 2007).

In the Palaeolithic, beads made from molluscs were desirable for body adornments (e.g., necklaces, headdresses), which likely varied due to decorative traditions of prehistoric human groups (Stiner, 2014). Although there is only rare evidence of shell bead arrangements from the Palaeolithic, we might expect beads strung in different arrangements to require differently placed piercings for the shells to hang according to a predetermined design. The evidence indicates that prehistoric people were adept at piercing holes in shells, but also made use of natural perforations when possible (Bar-Yosef Mayer, Vandermeersch & Bar-Yosef, 2009), suggesting that perforation location may also have varied based on opportunistic natural hole placements.

People also appear to have preferred mollusc species with vivid markings and that vary in size and shape (Stiner, 2014). Inter-specific morphological differences in shell size and shape also encompass variations in shell thickness that impacts the ease with which a piercing can be made. All these factors have influenced the attraction of humans to particular species of mollusc and likely contributed to the variability of hole placement in shells, hence, the assumption that humans pierce shells in consistent places, may not be borne out (D’Errico et al., 2005; Kuhn et al., 2009; Stiner, Kuhn & Güleç, 2013).

Furthermore, animal predation on mollusc populations is a widespread phenomenon (Quensen & Woodruff, 1997; Rosin et al., 2011). Such behaviours have been observed for many hole-boring predators, such as naticids, muricids, octopuses, crabs and birds (Grey, Lelievre & Boulding, 2005; Grey, 2005; Rosin et al., 2011; Li, Young & Zhan, 2011). Moreover, predators can be specific in where they attack molluscs because shell strength and location of internal organs can be important in prey selection (Hagadorn & Boyajian, 1997; Dodge & Scheel, 1999; Rosin et al., 2013). For instance, birds usually choose to perforate the part of the gastropod shell near the apex, which is less resistant to crushing than, for example, the labium (Rosin et al., 2013). In contrast, octopuses and predatory snails choose areas close to the bivalve umbo, which tends to be thicker than other areas of the shell, but is near to the heart. This strategy appears to be a compromise between the time taken to pierce the shell and the effectiveness of the injected toxin (Dodge & Scheel, 1999).

Recent evidence also indicates that holes in shells can be made without the action of predators or humans. In a set of shell-rolling experiments that imitate the action of the waves and tides, Gorzelak and co-workers (2013) showed that abrasive action of rolling shells together can create holes in predictable locations that coincide with the holes of predators. It is therefore possible that abrasion may also imitate human actions.

Considering that pierced shells can not only be produced by humans for making Palaeolithic jewellery, but can also be produced by natural processes, we examined if the range of variability of hole location in shells made by humans is less than the variability in hole location in shells made by non-human animals (Stiner, 1999; Bouzouggar et al., 2007). We discuss these findings with a focus on the actions of mollusc predators, and consider factors researchers might want to consider when interpreting shells recovered from archaeological contexts.

## Materials & Methods

The first part of this research assessed the variability of hole placement in shell beads made by humans. For this purpose we reviewed information within 316 publications including articles, PhD theses and chapters in books about malacological findings in archaeological contexts (Supplemental Information 3). Most of the gathered literature was written in English, with only a few papers published in other language (e.g., French or Spanish). We searched for these using Google Scholar and SCOPUS, and using keywords such as: shell beads, pierced shells, beads, shells, mollusc, Gastropoda, bivalves, pendant, shell midden, ornaments, shell ornaments, predators. Once the publications were selected, their references (backward search) and citation records (forward search) were analysed to find other articles that could provide relevant data (Table 1). Gathered literature was published between the 1966 and the first quarter of 2015. From these articles we selected papers which contained figures and information about the perforated shells. Related articles, which included the same figures of shells, were rejected. This approach ensured that site data was only assessed once (i.e., data were not replicated). We were able to select 79 papers from 316 gathered scientific articles.

**Table 1:** Hole assessment in shell beads from the archaeological sites.

Mollusc species (Class)	Country	Site (*N*)	Date	References	HT (*n*)
*Acanthocardia tuberculata* (Bivalvia)	Cyprus	Shillourokambus (1)	9.000–8.000 BP	Serrand, Vigne & Guilaine (2005)	9 (1)
	France	Balauzerie (1)	40.000–28.000 BP	Barge (1983)	9 (1)
		Régismont (1)	40.000–28.000 BP	Fiocchi (1998)	9 (1)
		Tournal (1)	40.000–28.000 BP	Fiocchi (1998)	9 (1)
	Italy	Fanciulli (1)	40.000–28.000 BP	Barge (1983)	9 (1)
		Riparo Mochi (1)	34.870–32.280 BP	Barge (1983)	9 (1)
	Spain	Cueva de los Aviones (1)	50.000 BP	Zilhão et al. (2010), Vanhaeren & D’Errico (2011)	9 (1)
		Cova de l’Or (4)	6.720–6.265 BP	Zilhão et al. (2010)	9 (4)
		Cova del Parpallo (2)	50.000–10.000 BP	Zilhão et al. (2010)	9 (1), 10 (1)
	Turkey	Üçağızlı (1)	41.000–39.000 BC	Stiner, Kuhn & Güleç (2013)	10 (1)
*Antalis* sp. (Scaphopoda)	Italy	Fumane (1)	41.000–38.000 BP	Bertola et al. (2013)	5 (1)
	Portugal	Vale Boi (4)	20.570–18.859 BP	Tátá et al. (2014)	5 (4)
	Spain	El Cuco (30)	29.000–22.000 ka	Gutiérrez-Zugasti & Cuenca-Solana (2013)	5 (30)
		Guilanya (5)	14.160–9.500 BP	Martinez-Moreno, Mora & Casanova (2010)	5 (5)
		Tito Bustillo (1)	18.000–10.000 ka	Avezuela (2014)	5 (1)
*Bolinus brandaris* (Gastropoda)	Cyprus	Shillourokambus (1)	9.000–8.000 BP	Serrand, Vigne & Guilaine (2005)	4 (1)
*Buccinum undatum* (Gastropoda)	Italy	Riparo Tagliente (1)	14.600–11.5000 BC	Fontana et al. (2009)	4 (1)
*Cerastoderma* sp. (Bivalvia)	Spain	Cova del Parpallo (8)	50.000–10.000 BP	Zilhão et al. (2010)	9 (4), 10 (4)
*Cerithium* sp. (Gastropoda)	Italy	Riparo Mochi (1)	34.870–32.280 BP	Kuhn & Stiner (1998)	7 (1)
		Riparo Tagliente (1)	14.600–11.5000 BC	Fontana et al. (2009)	6 (1)
	Jordan	Wadi Mataha (1)	15.579–11.042 BP	Janetski & Bar-Yosef (2005)	2 (1)
*Chlamys* sp. (Bivalvia)	Italy	Riparo Mochi (1)	34.870–32.280 BP	Kuhn & Stiner (1998)	10 (1)
*Clanculus corallines* (Gastropoda)	Greece	Klisoura (1)	41.000–38.000 BP	Stiner (2010)	6 (1)
	Italy	Cala (1)	40.000–28.000 BP	Fiocchi (1998)	4 (1)
		Riparo Mochi (3)	34.870–32.280 BP	Kuhn & Stiner (1998)	4 (3)
*Columbella* sp. (Gastropoda)	Austria	Krems-Hundsteig (10)	40,000–28,000 BP	Fiocchi (1998), Wild et al. (2008)	2 (5), 3 (5)
	Croatia	Zala cave (N10)	11.070–10.500 BP	Komšo & Vukosavljević (2011)	3 (4), 4 (5), 7 (1)
	Cyprus	Shillourokambus (3)	9.000–8.000 BP	Serrand, Vigne & Guilaine (2005)	4 (3)
	Greece	Klisoura (1)	41.000–38.000 BP	Stiner (2010)	4 (1)
	Italy	Cala (1)	40.000–28.000 BP	Fiocchi (1998)	2 (1)
		Grotta di Pozzo (2)	85.000–60.000 BP	Mussi et al. (2000)	4 (2)
		Riparo Biarzo (16)	12.000–5.600 BP	Cristiani (2012)	3 (1), 4 (7), 6 (8)
		Riparo Tagliente (1)	14.600–11.5000 BC	Fontana et al. (2009)	4 (1)
	Near East	Ksar Akil (6)	41.000–39.000 BC	Inizan (1978), Douka (2013)	3 (2), 7 (3), 4 (1), 11 (1)
		Sefunim (1)	41.000–15.000 BP	Bar-Yosef (1996a)	4 (1)
	Russia	Kostienki 1 (1)	36.500–32.600 BP	Sinitsyn (1993)	2 (1)
	Spain	Botiquería de Los Moros (6)	6.000–4.000 BP	Álvarez-Fernández (2010)	4 (2), 8 (3), 11 (1)
	Turkey	Pınarbaşı (3)	8.5000–8.000 BC	Baysal (2013)	8 (1)
*Conus* sp. (Gastropoda)	Australia	Mandu Mandu Creek rock-shelter (1)	35.200–30.900 BP	Morse (1993), Balme & Morse (2006)	8 (1)
	Cyprus	Shillourokambus (2)	9.000–8.000 BP	Serrand, Vigne & Guilaine (2005)	8 (2)
	Italy	Cala (1)	40.000–28.000 BP	Fiocchi (1998)	4 (1)
		Riparo Mochi (1)	34.870–32.280 BP	Kuhn & Stiner (1998), Stiner (1999)	2 (1)
	Oman	Sumhuram (1)	4.000–1.000 BP	Wilkens (2005)	8 (1)
	Turkey	Üçağızlı (1)	41.000–39.000 BC	Stiner, Kuhn & Güleç (2013)	8 (1)
*Cyclope* sp. (Gastropoda)	France	Abri Peyrony (13)	40.000–28.000 BP	Vanhaeren & D’Errico (2011)	1 (1), 3 (6), 4 (1), 11 (3)
		Rothschild (1)	40.000–28.000 BP	Zilhão (2007)	1 (1)
	Germany	Andernach-Martinsberg (4)	13.200–12.820 BP	Langley & Street (2013)	1 (2), 3 (2)
	Greece	Klisoura (1)	41.000–38.000 BP	Stiner (2010)	6 (1)
	Italy	Fumane (1)	41.000–38.000 BP	Bertola et al. (2013)	4 (1)
		Riparo Biarzo (1)	9.000–7.000 BP	Cristiani (2012)	1 (1)
		Riparo Mochi (25)	34.870–32.280 BP	Kuhn & Stiner (1998), Stiner (1999)	1 (25)
		Riparo Tagliente (2)	14.600–11.5000 BC	Fontana et al. (2009)	1 (2)
	Spain	Cingle Vermell (1)	9.760 BP	Oliva & Yll (2010)	1 (1)
		La Pena de Estebanvela (4)	12.000–9.000 BP	Avezuela (2014)	1 (2), 4 (2)
		Nerja Cave (2)	25.000–21.000 BP	Jordá Pardo et al. (2010)	1 (2)
		Tito Bustillo (1)	18.000–10.000 ka	Avezuela (2014)	1 (1)
*Cymatium parthenopeum* (Gastropoda)	Cyprus	Shillourokambus (1)	9.000–8.000 BP	Serrand, Vigne & Guilaine (2005)	6 (1)
*Cypraea* sp. (Gastropoda)	India	Deccan region (3)	2.300–900 BC	Deshpande-Mukherjee (2005)	5 (3)
*Dentalium* sp. (Scaphopoda)	Austria	Krems-Hundsteig (1)	40.000–28.000 BP	Fiocchi (1998), Neugebauer-Maresch (1999)	5 (1)
		Langmannersdorf (5)	40.000–28.000 BP	Hahn (1972)	5 (5)
		Senftenberg (1)	40.000–28.000 BP	Hahn (1972)	5 (1)
		Willendorf (1)	28.000–22.000 BP	Kozłowski (1996)	5 (1)
	Cyprus	Shillourokambus (2)	9.000–8.000 BP	Serrand, Vigne & Guilaine (2005)	5 (2)
	France	Abri Peyrony (600)	40.000–28.000 BP	Vanhaeren & D’Errico (2011)	5 (600)
		Blanchard (2)	34.000–32.000 BP	Taborin (1993)	5 (2)
		Caminade Est (1)	37.200–32.140 BP	Taborin (1993)	5 (1)
		Castanet (1)	34.000–32.000 BP	Taborin (1993)	5 (1)
		Cellier (1)	40.000–28.000 BP	Taborin (1993)	5 (1)
		Laouza (1)	40.000–28.000 BP	Fiocchi (1998)	5 (1)
		Pecheurs (1)	28.000–22.000 BP	Barge (1983)	5 (1)
		Rochette (1)	40.000–28.000 BP	Movius (1995)	5 (1)
		Rothschild (2)	40.000–28.000 BP	Zilhão (2011)	5 (2)
		Saint-Germain-la-Rivière (1)	15.570 BP	Vanhaeren & D’Errico (2005)	5 (1)
		Salpetriere (1)	22.000–18.000 BP	Barge (1983)	5 (1)
		Tournal (1)	40.000–28.000 BP	Fiocchi (1998)	5 (1)
		Tuto de Camalhot (1)	40.000–28.000 BP	Taborin (1993)	5 (1)
		Vachons (1)	40.000–28.000 BP	Taborin (1993)	5 (1)
	Greece	Klisoura (1)	41.000–38.000 BP	Stiner (2010)	5 (1)
	Italy	Cala (1)	40.000–28.000 BP	Fiocchi (1998)	5 (1)
		Grotta del Cavallo (1)	31.000–21.000 BP	Cesnola & Mallegni (1996)	5 (1)
		Grotta di Pozzo (4)	85.000–60.000 BP	Mussi et al. (2000)	5 (4)
		Fanciulli (1)	40.000–28.000 BP	Barge (1983)	5 (1)
		Fumane (1)	41.000–38.000 BP	Fiocchi (1998)	5 (1)
		Riparo Mochi (3)	34.870–32.280 BP	Kuhn & Stiner (1998)	5 (3)
		Riparo Tagliente (1)	14.600–11.5000 BC	Fontana et al. (2009)	5 (1)
	Jordan	Wadi Mataha (6)	15.579–11.042 BP	Janetski & Bar-Yosef (2005)	5 (6)
	Near East	Ksar Akil (1)	41.000–39.000 BP	Douka (2013)	5 (1)
	Spain	Beneito (1)	40.000–28.000 BP	Soler-Major (2001)	5 (1)
		Cingle Vermell (1)	9.760 BP	Oliva & Yll (2010)	5 (1)
		Cova del Parco (2)	13.175–12.460 BP	Estrada et al. (2010), Mangado et al. (2010)	5 (2)
		Cova del Reclau Viver (53)	39.000–29.000 BP	Avezuela Aristu & Álvarez-Fernández (2012)	5 (53)
		Nerja Cave (2)	25.000–21.000 BP	Jordá Pardo et al. (2010)	5 (2)
		Roc del Migdia (4)	8.800–8.190 BP	Oliva & Yll (2010)	5 (4)
	Turkey	Çatalhöyük (5)	7.200–6.000 BP	Bar-Yosef Mayer, Gümüs & Islamoglu (2010)	5 (5)
		Pınarbaşı (2)	8.5000–8.000 BC	Baysal (2013)	5 (2)
		Boncuklu Höyük (3)	9.000–8.000 BC	Baysal (2013)	5 (3)
*Engina mendicaria* (Gastropoda)	Eritrea	Red Sea Coast (3)	7.330–5.385 BP	Bar-Yosef-Mayer & Beyin (2009)	4 (3)
*Euthria cornea* (Gastropoda)	Turkey	Üçağızlı (1)	41.000–39.000 BC	Stiner, Kuhn & Güleç (2013)	4 (1)
*Gibbula* sp. (Gastropoda)	Italy	Fumane (2)	41.000–38.000 BP	Bertola et al. (2013)	4 (2)
	Turkey	Üçağızlı (1)	41.000–39.000 BC	Stiner, Kuhn & Güleç (2013)	4 (1)
*Glycymeris* sp. (Bivalvia)	Cyprus	Shillourokambus (1)	9.000–8.000 BP	Serrand, Vigne & Guilaine (2005)	9 (1)
	France	Figuier (1)	40.000–28.000 BP	Taborin (1993)	9 (1)
	Israel	Qafzeh cave (1)	90.000 y BP	Taborin (1993)	9 (1)
	Italy	Fumane (6)	41.000–38.000 BP	Vanhaeren & D’Errico (2011), Bertola et al. (2013)	9 (6)
	Portugal	Gruta do Caldeirao (1)	6.500–5.800 BP	Zilhão et al. (2010)	4 (1)
	Spain	Cova de l’Or (16)	6.720–6.265 BP	Zilhão et al. (2010)	9 (16)
			Cova del Parpallo (7)	50.000–10.000 BP	Zilhão et al. (2010)	9 (7)
		Cueva de los Aviones (2)	50.000 BP	Zilhão et al. (2010)	9 (2)
*Hexaplex trunculus* (Gastropoda)	Cyprus	Shillourokambus (2)	9.000–8.000 BP	Serrand, Vigne & Guilaine (2005)	4 (2)
*Homalopoma sanguineum* (Gastropoda)	Germany	Andernach-Martinsberg (54)	13.200–12.820 BP	Álvarez-Fernández (2009), Langley & Street (2013)	1 (1), 3 (1), 4 (21), 8 (8)
	Greece	Klisoura (1)	41.000–38.000 BP	Stiner (2010)	6 (1)
	Italy	Fumane (1)	41.000–38.000 BP	Bertola et al. (2013)	4 (1)
	Spain	Cova del Parco (1)	13.175–12.460 BP	Mangado et al. (2010)	11 (1)
		Tito Bustillo (1)	18.000–10.000 ka	Álvarez Fernández (2006)	4 (1), 8 (1)
*Lithoglyphus* sp. (Gastropoda)	Croatia	Pupićina Cave (1)	11.070–10.500 BP	Komšo & Vukosavljević (2011)	6 (1)
		Zala cave (20)	11.070–10.500 BP	Komšo & Vukosavljević (2011)	1 (9), 4 (11)
	Italy	Riparo Biarzo (7)	12.000–7.000 BP	Cristiani (2012)	3 (3), 7 (4)
*Littorina littorea* (Gastropoda)	France	Gargas Cave (1)	26.910–23.590 BP	Juan-Foucher & Foucher (2008)	1 (1)
		Hautes-Pyrénées (3)	21.000–10.000 BP	Cattelain (2012)	1 (1), 2 (1), 4 (1)
	Spain	El Cuco (1)	29.000–22.000 ka	Gutiérrez-Zugasti & Cuenca-Solana (2013)	2 (1)
		Tito Bustillo (1)	18.000–10.000 ka	Álvarez Fernández (2006)	4 (1)
*Littorina obtusata* (Gastropoda)	France	Gargas Cave (2)	26.910–23.590 BP	Juan-Foucher & Foucher (2008)	1 (1), 4 (1)
		Hautes-Pyrénées (97)	10.000–6.000 BP	Cattelain (2012)	1 (1), 2 (10), 4 (11)
		Rothschild (1)	40.000–28.000 BP	Zilhão (2011)	1 (1)
	Spain	Cueto de La Mina (1)	50.000–10.000 BP	Cáceres, Marcos & Diez (2008)	4 (1)
		El Cuco (2)	29.000–22.000 ka	Gutiérrez-Zugasti & Cuenca-Solana (2013)	2 (1), 3 (1)
		El Horno (2)	12.862–12.481 BP	Fano & Álvarez-Fernández (2010)	1 (2)
		La Garma A (10)	29.000–22.000 ka	Álvarez-Fernández (2007)	1 (1), 4 (4), 8 (2)
		Maltravieso cave (1)	40.000–10.000 BP	Rodríguez Hidalgo et al. (2010)	1 (1)
		Nerja Cave (2)	25.000–21.000 BP	Jordá Pardo et al. (2010)	1 (1), 4 (1)
*Littorina* sp. (Gastropoda)	Italy	Fumane (1)	41.000–38.000 BP	Bertola et al. (2013)	4 (1)
		Riparo Mochi (1)	34.870–32.280 BP	Stiner (2010)	8 (1)
	France	Saint-Jean-De-Verges (6)	40.000–28.000 BP	Vezian & Vezian (1966)	1 (2), 4 (2)
	Portugal	Vale Boi (9)	20.570–18.859 BP	Tátá et al. (2014)	1 (2), 4 (7), 8 (3)
	South Africa	Sibudu Cave Middle Stone (3)	70.000–60.000 BP	D’Errico, Vanhaeren & Wadley (2008)	1 (2), 4 (1)
*Melanopsis* sp. (Gastropoda)	Turkey	Üçağızlı (1)	41.000–39.000 BC	Stiner, Kuhn & Güleç (2013)	3 (1)
*Mitra corniculata* (Gastropoda)	Italy	Riparo Mochi (1)	34.870–32.280 BP	Kuhn & Stiner (1998)	4 (1)
*Monodonta* sp. (Gastropoda)	Greece	Klisoura (1)	41.000–38.000 BP	Stiner (2010)	4 (1)
*Nassarius circumcintus* (Gastropoda)	Spain	Moroccan cave (1)	83.000–60.000 BP	D’Errico et al. (2009)	4 (1)
	Italy	Fumane (1)	41.000–38.000 BP	Bertola et al. (2013)	4 (1)
		Riparo Tagliente (1)	14.600–11.5000 BC	Fontana et al. (2009)	4 (1)
*Nassarius gibbosulus* (Gastropoda)	Algeria	Oued Djebbanna (1)	35.000 BP	Vanhaeren & D’Errico (2006), Douka (2013)	2 (1)
	Cyprus	Shillourokambus (6)	9.000–8.000 BP	Serrand, Vigne & Guilaine (2005)	6 (6)
	France	Blanchard (1)	34.000–32.000 BP	Taborin (1993)	4 (1)
		Rothschild (1)	40.000–28.000 BP	Zilhão (2011)	4 (1), 11 (1)
	Israel	Skhul (2)	110.000 BP	Vanhaeren & D’Errico (2006)	1 (1), 2 (1)
	Italy	Fumane (1)	41.000–38.000 BP	Vanhaeren & D’Errico (2011)	3 (1)
		Riparo Mochi (1)	40.000–28.000 BP	Kuhn & Stiner (1998), Stiner (1999)	4 (1)
	Morocco	Grotte des Contrebandiers (1)	40.000–12.500 BP	Vanhaeren & D’Errico (2011)	4 (1)
		Grotte des Pigeons, Taforalt (13)	83.000–81.000 BP	Vanhaeren & D’Errico (2011), Elias (2012), Douka (2013)	1 (1), 2 (5), 3 (1), 4 (4), 6 (1), 11 (1)
	Near East	Ksar Akil (2)	41.000–39.000 BP	Douka et al. (2013)	1 (1), 4 (1)
		Sefunim (1)	41.000–15.000 BP	Bar-Yosef (1996b)	4 (1)
	Spain	Moroccan cave (17)	83.000–60.000 BP	D’Errico et al. (2009)	2 (4), 3 (3), 4 (8), 11 (2)
	Turkey	Üçağızlı (11)	41.000–39.000 BC	Kuhn et al. (2001), Stiner, Kuhn & Güleç (2013)	1 (1), 2 (1), 3 (2), 4 (6), 7 (2), 8 (1)
*Nassarius incrassatus* (Gastropoda)	Italy	Fumane (2)	41.000–38.000 BP	Vanhaeren & D’Errico (2011), Bertola et al. (2013)	2 (2)
		Riparo Mochi (1)	34.870–32.280 BP	Kuhn & Stiner (1998)	4 (1)
		Riparo Tagliente (1)	14.600–11.5000 BC	Fontana et al. (2009)	4 (1)
	Spain	Cingle Vermell (1)	9.760 BP	Oliva & Yll (2010)	1 (1)
		El Horno (1)	12.862–12.481 BP	Fano & Álvarez-Fernández (2010)	3 (1)
		Roc del Migdia (7)	8.800–8.190 BP	Oliva & Yll (2010)	4 (7)
*Nassarius kraussianus* (Gastropoda)	South Africa	Blombos Cave (2)	78.000–75.600 BP	D’Errico et al. (2005), Douka (2013), Vanhaeren et al. (2013)	1 (1), 3 (1)
		Border Cave (2)	44.000–22.000 BP	D’Errico et al. (2012)	3 (1), 4 (1), 11 (2)
*Nassarius mutabilis* (Gastropoda)	France	Rothschild (1)	40.000–28.000 BP	Zilhão (2011)	4 (1)
	Italy	Fumane (2)	41.000–38.000 BP	Vanhaeren & D’Errico (2011), Bertola et al. (2013)	2 (1), 4 (1)
	Spain	Tito Bustillo (1)	18.000–10.000 ka	Álvarez Fernández (2006)	1 (1),
	Turkey	Üçağızlı (11)	41.000–39.000 BC	Stiner, Kuhn & Güleç (2013)	3 (9), 11 (5)
*Nassarius reticulates* (Gastropoda)	Italy	Fumane (1)	41.000–38.000 BP	Bertola et al. (2013)	3 (1)
	France	Rothschild (1)	40.000–28.000 BP	Zilhão (2011)	3 (1)
	Russia	Mezmaiskaya Cave (1)	36.000–28.510 BP	Golovanova, Doronichev & Cleghorn (2010)	2 (1), 11 (1)
	Spain	El Horno (1)	12.862–12.481 BP	Fano & Álvarez-Fernández (2010)	3 (1)
		Tito Bustillo (1)	18.000–10.000 ka	Avezuela (2014)	1 (1)
*Nassarius* sp. (Gastropoda)	Italy	Fumane (1)	41.000–38.000 BP	Bertola et al. (2013)	4 (1)
		Riparo Mochi (1)	34.870–32.280 BP	Kuhn & Stiner (1998)	4 (1)
	Jordan	Wadi Mataha (2)	15.579–11.042 BP	Janetski & Bar-Yosef (2005)	3 (2)
	Morocco	Grotte des Contrebandiers (1)	40.000–12.500 BP	D’Errico et al. (2009)	4 (1), 11 (1)
	Spain	Cova del Parco (1)	13.175–12.460 BP	Mangado et al. (2010)	4 (1)
	Turkey	Boncuklu Höyük (1)	9.000–8.000 BC	Baysal (2013)	11 (1)
		Pınarbaşı (2)	8.5000–8.000 BC	Baysal (2013)	4 (2)
*Natica* sp. (Gastropoda)	Italy	Fumane (1)	41.000–38.000 BP	Vanhaeren & D’Errico (2011)	3 (1)
	Spain	Cova del Parco (1)	13.175–12.460 BP	Mangado et al. (2010)	3 (1)
*Naticarius* sp. (Gastropoda)	Turkey	Üçağızlı (2)	41.000–39.000 BC	Stiner, Kuhn & Güleç (2013)	1 (2), 8 (1)
*Neritina picta* (Gastropoda)	France	Hautes-Pyrénées (1)	21.000–10.000 BP	Cattelain (2012)	4 (1)
		Abri Peyrony (99)	40.000–28.000 BP	Vanhaeren & D’Errico (2011)	1 (1), 4 (54)
		Gargas Cave (1)	26.910–23.590 BP	Juan-Foucher & Foucher (2008)	1 (1)
*Nucella lapillus* (Gastropoda)	France	Hautes-Pyrénées (2)	21.000–10.000 BP	Cattelain (2012)	1 (1), 11 (1), 4 (1)
		Gargas Cave (2)	26.910–23.590 BP	Juan-Foucher & Foucher (2008)	1 (2), 11 (1)
		Rothschild (1)	40.000–28.000 BP	Zilhao (2010)	4 (1)
		Saint-Germain-la-Rivière (1)	15.570 BP	Vanhaeren & D’Errico (2005)	4 (1)
	Greece	Klisoura (1)	41.000–38.000 BP	Stiner (2010)	4 (1)
	Spain	La Garma A (1)	29.000–22.000 ka	Avezuela Aristu & Álvarez-Fernández (2012)	4 (1), 8 (1), 11 (1)
*Ocinebrina edwardsii* (Gastropoda)	Spain	Cueto de la Mina (1)	18.000–10.000 ka	Sella (1916)	2 (1)
	Italy	Riparo Mochi (7)	34.870–32.280 BP	Kuhn & Stiner (1998), Stiner (1999)	4 (7)
*Oliva bulbosa* (Gastropoda)	Oman	Sumhuram (9)	4.000–1.000 BP	Wilkens (2005)	8 (9)
*Patella vulgata* (Gastropoda)	France	Hautes-Pyrénées (3)	21.000–10.000 BP	Cattelain (2012)	10 (3)
	Spain	La Garma A (1)	29.000–22.000 ka	Avezuela Aristu & Álvarez-Fernández (2012)	10 (1)
		Maltravieso cave (1)	40.000–10.000 BP	Rodríguez Hidalgo et al. (2010)	10 (1)
*Pecten* sp. (Bivalvia)	France	Gargas Cave (1)	26.910–23.590 BP	Juan-Foucher & Foucher (2008)	10 (1)
		Rothschild (1)	40.000–28.000 BP	Zilhão (2007)	1 (1)
	Spain	Cueva Anton (1)	38.440–36.810 BP	Zilhão et al. (2010)	4 (1)
		Riparo Mochi (1)	40.000–28.000 BP	Stiner (1999)	4 (1)
*Persicula terveriana* (Gastropoda)	Eritrea	Red Sea Coast (1)	7.330–5.385 BP	Bar-Yosef-Mayer & Beyin (2009)	4 (1)
*Pirenella plicata* (Gastropoda)	France	Hautes-Pyrénées (1)	21.000–10.000 BP	Cattelain (2012)	2 (1)
*Theodoxus fluviatilis* (Gastropoda)	France	Gargas Cave (1)	26.910–23.590 BP	Juan-Foucher & Foucher (2008)	1 (1)
		Hautes-Pyrénées (1)	21.000–10.000 BP	Cattelain (2012)	1 (1)
		Rothschild (1)	40.000–28.000 BP	Zilhão (2007)	1 (1)
	Portugal	Vale Boi (5)	20.570–18.859 BP	Tátá et al. (2014)	4 (5)
	Russia	Kostienki 14 (4)	36.500–32.600 BP	Sinitsyn (2003)	2 (2), 3 (1)
	Spain	Cova del Parco (2)	13.175–12.460 BP	Estrada et al. (2010), Mangado et al. (2010)	1 (1) 4 (1)
		Nerja Cave (4)	25.000–21.000 BP	Jordá Pardo et al. (2010)	4 (4)
*Theodoxus* sp. (Gastropoda)	Greece	Klisoura (3)	41.000–38.000 BP	Stiner (2010)	6 (3)
	Italy	Riparo Biarzo (2)	12.000–7.000 BP	Cristiani (2012)	1 (1), 8 (2)
	Turkey	Üçağızlı (1)	41.000–39.000 BC	Stiner, Kuhn & Güleç (2013)	4 (1)
*Trivia* sp. (Gastropoda)	France	Gargas Cave (3)	26.910–23.590 BP	Juan-Foucher & Foucher (2008)	3 (3), 11 (2)
		Hautes-Pyrénées (2)	21.000–10.000 BP	Cattelain (2012)	1 (2)
		Rothschild (1)	40.000–28.000 BP	Zilhão (2011)	3 (1)
		Saint-Germain-la-Rivière (3)	15.570 BP	Vanhaeren & D’Errico (2005)	5 (3)
	Italy	Fumane (1)	41.000–38.000 BP	Bertola et al. (2013)	11 (1)
		Riparo Mochi (1)	34.870–32.280 BP	Kuhn & Stiner (1998), Stiner (1999)	3 (1)
	Portugal	Vale Boi (4)	20.570–18.859 BP	Tátá et al. (2014)	5 (1), 8 (3)
	Spain	Cingle Vermell (2)	9.760 BP	Oliva & Yll (2010)	5 (2)
		El Horno (1)	12.862–12.481 BP	Fano & Álvarez-Fernández (2010)	5 (1)
		La Fragua (1)	12.960 BP	Zugasti (2010.)	5 (1)
		La Pena de Estebanvela (4)	12.000–9.000 BP	Avezuela (2014)	5 (2)
		Los Canes (6)	7.930–7.580 BP	Álvarez-Fernández (2010)	3 (6)
		Nerja Cave (1)	25.000–21.000 BP	Jordá Pardo et al. (2010)	3 (1)
		Tito Bustillo (1)	18.000–10.000 ka	Avezuela (2014)	5 (1)
*Trophon muricatus* (Gastropoda)	Russia	Mezmaiskaya Cave (1)	36.000–28.510 BP	Golovanova, Doronichev & Cleghorn (2010)	11 (1)
*Turritella* sp. (Gastropoda)	Italy	Fumane (1)	41.000–38.000 BP	Bertola et al. (2013), Taborin (1993)	2 (1)
	France	Abri Peyrony (25)	40.000–28.000 BP	Vanhaeren & D’Errico (2011)	2 (6), 3 (19)
	Spain	Cova del Reclau Viver (1)	39.000–29.000 BP	Avezuela Aristu & Álvarez-Fernández (2012)	3 (1), 11 (1)
		El Horno (4)	18.000–10.000 ka	Álvarez Fernández (2006)	2 (4)

**Notes.**

*N* the number of mollusc species *n* the number of shells with appropriate hole type HT hole type

**Figure 1:**
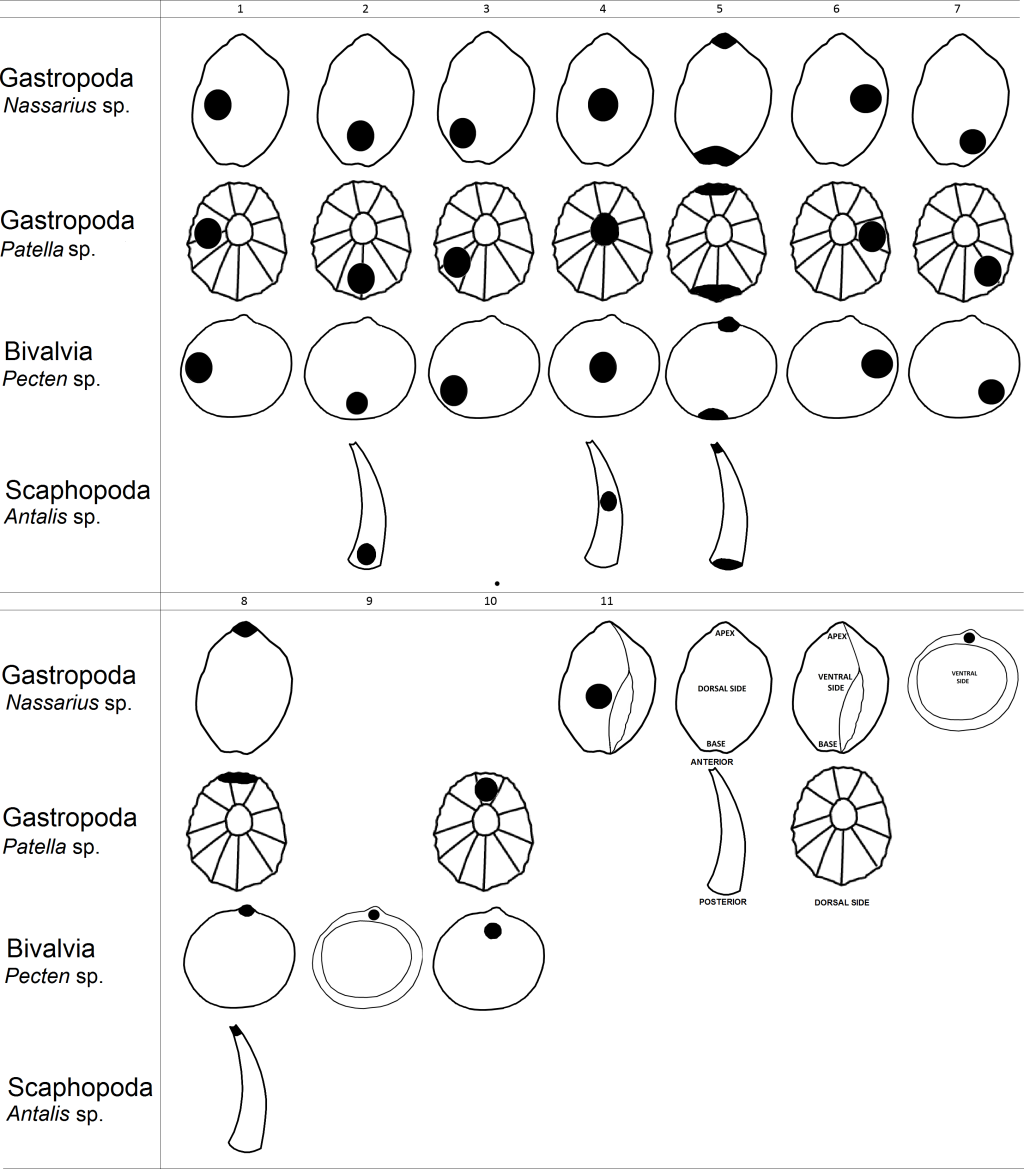
Graphical representation of hole location types in shells.

Information from all 79 papers was assessed for the following information: (1) mollusc species from which the shell beads were made; (2) name and country of the archaeological site where the perforated shells were found; (3) period from which the shell beads came; (4) hole location in the shell beads (Table 1). We made the assumption that analyses performed by experts correctly interpreted holes in shells as human made (i.e., the pierced shells were not predator-made intrusions).

Next, we created a classification of hole location in shell beads which helped us analyse gathered data from the literature (Fig. 1). As an example of shell shape we used species from genus *Nassarius*, *Patella, Pecten* and *Antalis* which are relatively common in the archaeological findings. Then, we assessed hole location in shells found in the archaeological literature (i.e., holes made by humans), based on the figures in each article. Our estimation was based on the figures within the publications, thus the analysis is not quantitative, but qualitative.

In the second part of the research we analysed variation of hole location in shells made by non-human animals. Thus, we searched for information on hole-making predators for each mollusc species recovered from each archaeological site with putative shell beads (Table 2). For this purpose, we gathered information on the location of holes in the shells made by mollusc predators from 54 scientific articles. To be clear, these articles were not associated with archaeological finds. Next, we assessed hole locations in shells made by hole-boring predators using the same classification that was used for the human-made holes in shell beads (Fig. 1). This part of the research was also based on the figures within the publications. We made the assumption that analyses by experts correctly interpreted holes in shells as naturally made and not made by humans (i.e., the shells were pierced by predators).

**Table 2:** Drilling predators for mollusc species used as a shell bead by prehistoric human groups.

Mollusc species recovered from archaeological sites (Class)	Predators class	Predator	References	HT (*n*)
*Acanthocardia tuberculata* (Bivalvia)	Gastropoda	*Naticarius hebraeus*	Calvet (1992)	4 (1), 9 (1)
*Antalis* sp. (Scaphopoda)	No data			
*Bolinus brandaris* (Gastropoda)	Cephalopoda	*Octopus vulgaris*	Nixon & Maconnachie (1988), Passini & Garassino (2012)	No data
	Gastropoda	Naticidae		
*Buccinum undatum* (Gastropoda)	Gastropoda	*Euspira macilenta*	Sawyer (2010)	No data
*Cerastoderma* sp. (Bivalvia)	Gastropoda	*Polinices pulchellus* *Hexaplex trunculus*	Kingsley-Smith, Richardson & Seed (2003), Morton, Peharda & Harper (2007)	1 (1), 4 (1), 10 (1)
*Cerithium* sp. (Gastropoda)	Gastropoda	*Euspira macilenta*	Turra, Denadai & Leite (2005), Sawyer & Zuschin (2010), Coleman (2010), Gorman, Sikinger & Turra (2015)	3 (1)
	Malacostraca	*Callinectes danae* *Eriphia gonagra* *Menippe node frons* *Panopeus occidentalis*		
*Chlamys* sp. (Bivalvia)	Asteroidea	*Pycnopodia helianthoides*	Guerrero & Reyment (1988), Farren & Donovan (2007), Chattopadhyay & Dutta (2013)	2 (1), 10 (1)
	Gastropoda	*Murex* sp. Naticidae		
*Clanculus corralinus* (Gastropoda)	No data			
*Columbella* sp. (Gastropoda)	Cephalopoda	*Octopus vulgaris*	Mather & O’Dor (1991)	No data
*Conus* sp. (Gastropoda)	Gastropoda	*Euspira macilenta*	Kohn & Arua (1999), Sawyer & Zuschin (2010)	4 (2), 8 (1), 11 (3)
*Cyclope* sp. (Gastropoda)	Asteroidea	*Astropecten* sp.	Baeta & Ramón (2013)	No data
*Cymatium parthenopeum* (Gastropoda)	No data			
*Cypraea* sp. (Gastropoda)	Cephalopoda	*Octopus vulgaris*	Nixon & Maconnachie (1988)	No data
*Dentalium* sp. (Scaphopoda)	Gastropoda	*Euspira macilenta* *Euspira obliquata* *Natica canrena* *Neverita duplicata* *Oichnus* sp.	Yochelson, Dockery & Wolf (1983), Sawyer & Zuschin (2010), Li, Young & Zhan (2011)	4 (25), 8 (1)
*Engina mendicaria* (Gastropoda)	No data			
*Euthria cornea* (Gastropoda)	Cephalopoda	*Octopus vulgaris*	Nixon & Maconnachie (1988), Nixon & Young (2003)	4 (2)
*Gibbula* sp. (Gastropoda)	Cephalopoda	*Octopus vulgaris*	Guerra & Nixon (1987), Mowles, Rundle & Cotton (2011)	4 (1)
	Malacostraca	*Carcinus maenas*		
*Glycymeris* sp. (Bivalvia)	Gastropoda	*Cryptonatica* sp. *Euspira* sp. *Glossaulax* sp. *Naticarius hebraeus*	Calvet (1992), Ramsay, Richardson & Kaiser (2001), Amano (2006), Sawyer & Zuschin (2010)	4 (1), 9 (1)
	Malacostraca	*Cancer pagurus*		
*Hexaplex trunculus* (Gastropoda)	Cephalopoda	*Octopus vugaris*	McQuaid (1994), Sawyer & Zuschin (2010), Passini & Garassino (2012)	4 (1), 11 (1)
	Gastropoda	Naticidae		
*Homalopoma sanguineum* (Gastropoda)	No data			
*Lithoglyphus* sp. (Gastropoda)	No data			
*Littorina littorea* (Gastropoda)	Asteroidea	*Pisastero straceaus* *Pycnopodia helianthoides*	Pechenik & Lewis (2000), Harley et al. (2013)	11 (1)
	Gastropoda	*Naticidae*		
*Littorina obtusata* (Gastropoda)	Aves	*Calidris canutus*	Alerstam, Gudmundsson & Johannesson (1992), Edgell et al. (2008), Edgell & Rochette (2009)	No data
	Malacostraca	*Carcinus maenas*		
*Littorina* sp. (Gastropoda)	Malacostraca	*Carcinus maenas*	Reimchen (1982)	1 (1), 4 (1)
*Melanopsis* sp. (Gastropoda)	Gastropoda	Gastropoda	Kowalewski, Rosa & Mancheno (2009)	No data
*Mitra corniculata* (Gastropoda)	Gastropoda	*Euspira macilenta*	Sawyer & Zuschin (2010), Cardoso & Dias Coelho (2012)	4 (1)
*Monodonta* sp. (Gastropoda)	Aves	Haematopodidae Laridae	Tongiorgi et al. (1981), Harris (1984)	4 (1)
	Gastropoda	*Ocinebrina edwardsi*		
*Nassarius circumcintus* *Nassarius gibbosulus* *Nassarius incrassatus* *Nassarius kraussianus* *Nassarius mutabilis* *Nassarius reticulates* *Nassarius* sp. (Gastropoda)	Malacostraca	*Carcinus maenas*	Stenzler & Atema (1977), Kohn & Arua (1999), Sawyer & Zuschin (2010)	8 (1), 11 (1)
	Gastropoda	*Euspira macilenta* *Lunatiaheros*, *Natica tecta*		
*Natica* sp. (Gastropoda)	Gastropoda	*Euspira macilenta* Naticidae, Muricidae	Arua (1989), Zlotnik (2001), Sawyer (2010), Das, Mondal & Bardhan (2013)	4 (1), 11 (37)
*Naticarius* sp. (Gastropoda)	Asteroidea	*Asterina sarasini*	Sawyer (2010)	No data
	Gastropoda	*Euspira macilenta*		
*Neritina picta* (Gastropoda)	Gastropoda	Acteocina Muricidae	Zagyvai & Demeter (2008)	4 (2), 8 (4), 11 (1)
	Polychaeta	Polychaeta		
*Nucella lapillus* (Gastropoda)	No data			
*Ocinebrina edwardsii* (Gastropoda)	No data			
*Oliva bulbosa* (Gastropoda)	Gastropoda	Naticidae	Kohn & Arua (1999), Passini & Garassino (2012)	11 (1)
*Patella vulgata* (Gastropoda)	Aves	*Haematopus ostralegus*	Coleman et al. (1999), Kohn & Arua (1999), Smith (2003), Silva et al. (2008), Silva et al. (2010), Sawyer (2010)	10 (1)
	Cephalopoda	*Octopus vulgaris*		
	Gastropoda	*Euspira macilenta*		
	Malacostraca	*Cancer pagurus,* *Carcinus maenas,* *Necora puber,* *Pachygrapsus marmoratus,*		
*Pecten* sp. (Bivalvia)	Asteroidea	*Asteria srubens* *Marthasterias glacialis*	Jonkers (2000), Sawyer (2010), Magnesen & Redmond (2011)	4 (2), 10 (1)
	Gastropoda	*Euspira macilenta*		
*Persicula terveriana* (Gastropoda)	Cephalopoda	*Octopus insularis*	Leite, Haimovici & Mather (2009)	No data
*Pirenella plicata* (Gastropoda)	Gastropoda	Naticidae	Taraschewski & Paperna (1982)	No data
*Theodoxus fluviatilis* (Gastropoda)	Aves	*Gallinula chloropus*	Blanco-Libreros & Arroyave-Rincón (2009)	1 (1), 4 (2)
	Malacostraca	*Macrobrachium* sp.		
*Theodoxus* sp. (Gastropoda)	Gastropoda	Muricidae	Arpad (1993), Zagyvai & Demeter (2008)	4 (2), 8 (4), 11 (1)
*Trivia* sp. (Gastropoda)	Asteroidea	*Asterina sarasini*	Sadhukhan & Raghunathan (2013)	No data
*Trophon muricatus* (Gastropoda)	Gastropoda	Naticidae	Gordillo & Archuby (2014)	11 (1)
*Turritella* sp. (Gastropoda)	Gastropoda	*Euspira macilenta* Naticidae Muricidae, *Odostomia* sp.	Allmon, Nieh & Norris (1990), Hagadorn & Boyajian (1997), Filipescu & Popa (2001), Sawyer (2010)	4 (3)

**Notes.**

*n* the number of shells with appropriate hole type HT hole type

Next, we analysed separately the types of hole location in shell beads made by human and non-human animals for normality by using the Shapiro Wilk test, and for homogeneity of variances by using Levene’s test; all were non-significant (*P* < 0.05). Analysed material did not fulfil the criterion of normality, thus data were log or square root transformed and tested again for normality (Sokal & Rohlf, 1981). After these transformations the data were still not normally distributed; we therefore used nonparametric sign test to analyse variation and differences in types of hole location between shells perforated by humans and non-human animals within mollusc species.

In order to analyse the strength of the differences between holes locations made by human and non-human animals we calculated size of the effect using the following equation: *d* = (*M*_1_ − *M*_2_)/SD_pooled_, where d is the Cohen-d index, M_1_ is the mean of the first group, M_2_ is the mean of the second group and SD_pooled_ is the pooled standard deviation (Cohen, 1988). To interpret *d* values we used the following criteria for effect sizes: *d*⩾0.1, small; *d*⩾0.3, medium; *d*⩾0.5, large (Cohen, 1988).

## Results

### Human made holes in shells

**Figure 2:**
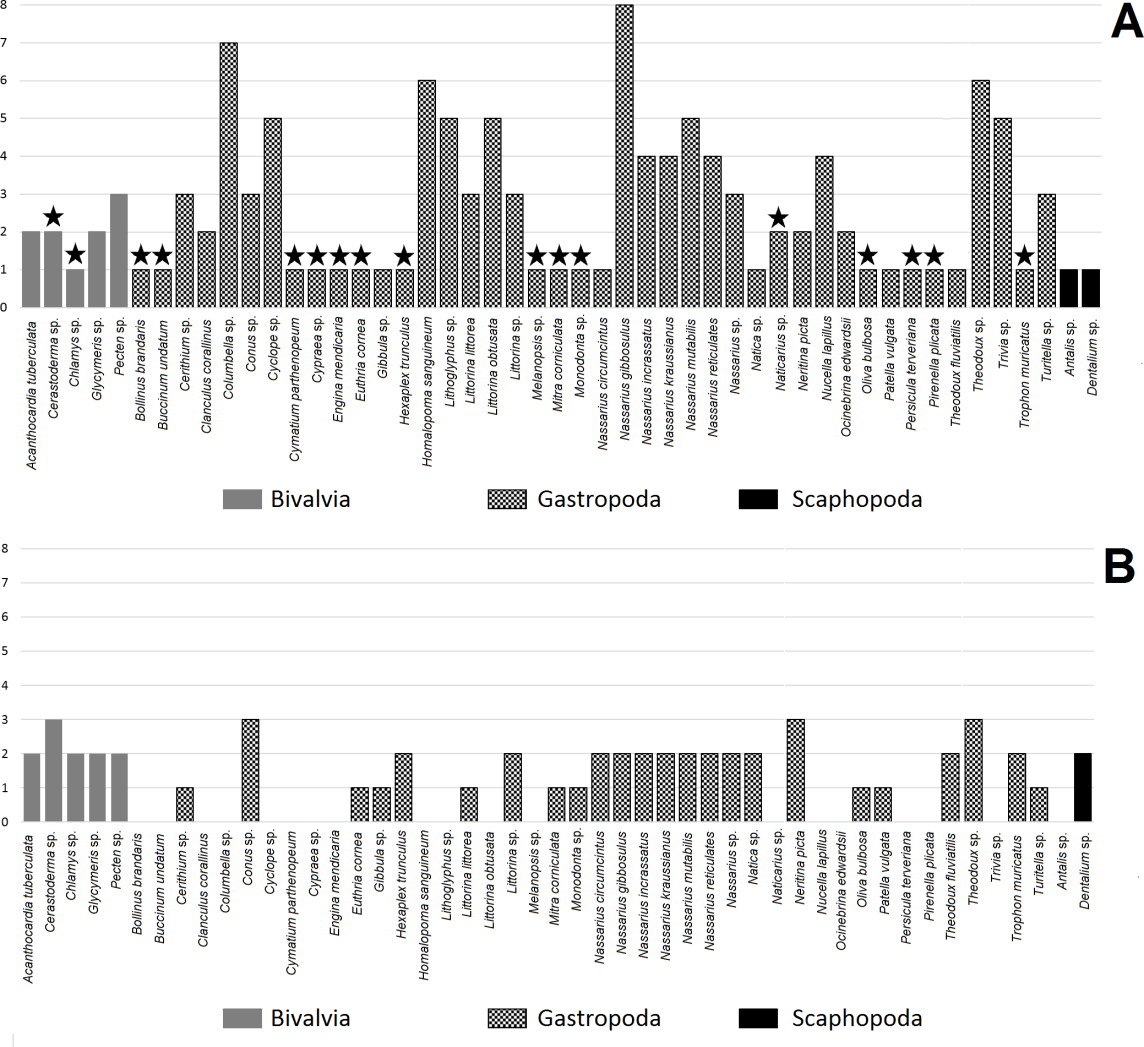
Number of hole location types in shell for mollusc species. A, made by humans; B, made by non-human animals. Star indicates taxon found at one archaeological site.

Table 1 shows hole assessment in 49 taxa of Mollusca perforated by humans from archaeological sites. Anthropogenically modified shells come from 21 countries of the Old World, with most archaeological sites located in Spain and France (Table 3). Twenty-seven taxa exhibited more than one type of hole location in their shells, while shells from 22 taxa were classified as having only one type of hole (Fig. 2A). However, 15 taxa from the latter group were recovered at one archaeological site alone.

**Table 3:** Summary class differences for hole location of Scaphopoda, Bivalvia and Gastropoda.

Country	Site	Bivalvia		Gastropoda		Scaphopoda		Summary	
		*N*	HT	*N*	HT	*N*	HT	*N*	*n*
Algeria	Oued Djebbanna			1	2			1	1
Australia	Mandu Mandu Creek rock-shelter			1	8			1	1
Austria	Krems-Hundsteig			1	2, 3	1	5	2	3
	Langmannersdorf					1	5	1	1
	Senftenberg					1	5	1	1
	Willendorf					1	5	1	1
Croatia	Pupićina Cave			1	6			1	1
	Zala cave			2	1, 3, 4, 7			2	4
Cyprus	Shillourokambus	2	9	6	4, 6, 8	1	5	9	5
Eritrea	Red Sea Coast			2	4			2	1
France	Abri Peyrony			3	1, 2, 3, 4, 11	1	5	4	6
	Balauzerie	1	9					1	1
	Blanchard			1	4	1	5	2	2
	Caminade Est					1	5	1	1
	Castanet					1	5	1	1
	Cellier					1	5	1	1
	Figuier	1	9					1	1
	Gargas Cave	1	10	7	1, 3, 4, 11			8	5
	Hautes-Pyrénées			8	1, 2, 4, 10, 11			8	5
	Laouza					1	5	1	1
	Pecheurs					1	5	1	1
	Régismont	1	9					1	1
	Rochette					1	5	1	1
	Rothschild	1	1	8	1, 3, 4, 11	1	5	10	5
	Saint-Germain-la-Rivière			2	4, 5	1	5	3	2
	Saint-Jean-De-Verges			1	1, 4			1	2
	Salpetriere					1	5	1	1
	Tournal	1	9			1	5	2	2
	Tuto de Camalhot					1	5	1	1
	Vachons					1	5	1	1
Germany	Andernach-Martinsberg			2	1, 3, 4, 8			2	4
Greece	Klisoura			7	4, 6	1	5	8	3
India	Deccan region			1	5			1	1
Israel	Qafzeh cave	1	9					1	1
	Skhul			1	1, 2			1	2
Italy	Cala			3	2, 4	1	5	4	3
	Fanciulli	1	9			1	5	2	2
	Fumane	1	9	13	2, 3, 4, 11	2	5	16	6
	Grotta del Cavallo					1	5	1	1
	Grotta di Pozzo			1	4	1	5	2	2
	Riparo Biarzo			4	1, 3, 4, 6, 7, 8			4	6
	Riparo Mochi	3	4, 9, 10	12	1, 2, 3, 4, 7, 8	1	5	16	9
	Riparo Tagliente			6	1, 4, 6	1	5	7	4
Jordan	Wadi Mataha			2	2, 3	1	5	3	3
Morocco	Grotte des Contrebandiers			2	4, 11			2	2
	Grotte des Pigeons, Taforalt			1	1, 2, 3, 4, 6, 11			1	6
Near East	Ksar Akil			2	1, 3, 4, 7, 11	1	5	3	6
	Sefunim			2	4			2	1
Oman	Sumhuram			2	8			2	1
Portugal	Gruta do Caldeirao	1	4					1	1
	Vale Boi			3	1, 4, 5, 8	1	5	4	4
Russia	Kostienki 14			2	2, 3			2	2
	Mezmaiskaya Cave			2	2, 11			2	2
South Africa	Blombos Cave			1	1, 3			1	2
	Border Cave			1	3, 4, 11			1	3
	Sibudu Cave Middle Stone			1	1, 4			1	2
Spain	Beneito					1	5	1	1
	Botiquería de Los Moros			1	4, 8, 11			1	3
	Cingle Vermell			3	1, 5	1	5	4	2
	Cova de l’Or	2	9					2	1
	Cova del Parco			4	1, 3, 4, 11	1	5	5	5
	Cova del Parpallo	3	9, 10					3	2
	Cova del Reclau Viver			1	3, 11	1	5	2	3
	Cueto de La Mina			2	2, 4			2	2
	Cueva Anton	1	4					1	1
	Cueva de los Aviones	2	9					2	1
	El Cuco			2	2, 3	1	5	3	3
	El Horno			5	1, 2, 3, 5			5	4
	Guilanya					1	5	1	1
	La Fragua			1	5			1	1
	La Garma A			3	1, 4, 8, 10, 11			3	5
	La Pena de Estebanvela			2	1, 4, 5			2	3
	Los Canes			1	3			1	1
	Maltravieso cave			2	1, 10			2	2
	Moroccan cave			2	2, 3, 4, 11			2	4
	Nerja Cave			4	1, 3, 4	1	5	5	4
	Roc del Migdia			1	4	1	5	2	2
	Tito Bustillo			6	1, 4, 5, 8	1	5	7	4
Turkey	Boncuklu Höyük			1	11	1	5	2	2
	Çatalhöyük					1	5	1	1
	Pınarbaşı			2	4, 8	1	5	3	3
	Üçağızlı	1	10	8	1, 2, 3, 4, 7, 8, 11			9	8

**Notes.**

*N* the number of mollusc species *n* the number of hole types HT hole type

The number of hole location types was diverse amongst mollusc species (Fig. 2A). Bivalves were more diverse in terms of hole location than Scaphopoda with most species exhibiting more than one type of hole location (Table 3). Gastropoda was the most numerous and diverse class in terms of hole location. *Nassarius gibbosulus* was the most variable species in terms of hole location among all archaeological sites (1, 2, 3, 4, 6, 7, 8 and 11) (Table 1; Fig. 2A). Moreover, shells of *Nassarius gibbosulus* from Üçağızlı (Turkey) and Grotte des Pigeons (Morocco) had the most variable hole location from single archaeological site (Table 3). Compared to other mollusc species, which are more frequently found at archaeological sites, but are less variable in terms of types of hole location, high variation in hole location in *Nassarius gibbosulus* does not appear to be a consequence of the relative abundance of this species.

Bivalves were the least common taxa found in archaeological assemblages and are characterised by hole locations 4, 9 and 10 (Table 3). Scaphopoda were more common than Bivalvia, but much less diverse in terms of types of hole location. Shell beads belonging to Scaphopoda had two holes, one in the anterior and the second one in the posterior of the shell (type number 5, Fig. 2A). In turn, gastropod shell beads were recovered at almost all archaeological sites and mostly showed more than one type of hole location.

Fumane (Italy) was the most diverse in context of number of gastropod species (13 taxa, Table 3), while Üçağızlı (Turkey) was characterised by the greatest number of types of hole location in gastropod shells at single archaeological site. Across all species, the most variable placed holes in shells were found at the site of Riparo Mochi (Italy) dated to the earliest Aurignacian (Kuhn & Stiner, 1998; Stiner, 1999).

### Predator made holes in shells

Table 2 shows that almost all mollusc taxa recovered from the analysed archaeological sites are preyed upon by hole-making predators (41 taxa). Among these taxa we were able to assess the hole location in the shells made by non-human animals in 30 mollusc species. Most of the assessed species exhibited two types of hole location in their shells, while only four species were classified as having three types of hole (Fig. 2B).

All species from the Bivalvia have predators that make holes in shells (Table 2). Species from this mollusc class are usually associated with more than one predator. Gastropoda is the class with the most numerous predators (nine species), then the Asteroidea (three species) and then Malacostraca with only one predator (Table 2). Almost all taxa from the Gastropoda have non-human predators that attack the prey by piercing holes in the mollusc’s shell.

Assessment of hole location types in shells made by predators was possible only for one Scaphopoda species (*Dentalium* sp., Table 2). *Dentalium* sp. can be attacked by five species of the Gastropoda and is pierced by gastropod predators in two places: in the middle or at the apex of the shell. Bivalvia is more diverse than Scaphopoda in terms of the placement of holes made by non-human predators. Gastropoda, Asteroidea and Malacostraca make holes in bivalves in the following locations: 1, 2, 3, 9 and 10 (Fig. 1). In turn, gastropods have predators that usually pierce holes in only one or two locations in particular species (Table 2, Fig. 2B).

### Comparison of hole locations made by humans and predators

The number of hole location types per scaphopod and bivalve species were higher for hole-boring predators than for anthropogenically modified shells. In turn, holes in gastropod shells made by humans were characterised by slightly greater variation than holes in shells pierced by non-human predators (Table 4). However, the sign test revealed no significant difference in types of hole location between shells perforated by humans and non-human animals within mollusc species (Table 4, *P* = 0.398). The result of the sign test was supported by value of the Cohen-d index. For differences between human made and non-human made holes in shell, the Cohen’s d value was small (*d* = 0.14, Table 4).

**Table 4:** Descriptive statistics and results of Kruskal–Wallis test for mollusc classes.

Class	*N*	Mean	SD	Min	Max	*N*	*P*	*Z*	*d*
*Human made holes in shells*						60	0.398	−0.845	0.14
Scaphopoda	2	1.00	0.00	1	1				
Bivalvia	5	2.00	0.63	1	3				
Gastropoda	42	2.72	1.93	1	8				
*Non-human made holes in shells*									
Scaphopoda	1	2.00	0.00	2	2				
Bivalvia	5	2.20	0.40	2	3				
Gastropoda	30	1.71	0.68	1	3				

**Notes.**

*N* the number of mollusc taxa SD standard deviation Min minimal number of hole location types at archaeological site Max maximal number of hole location types at archaeological site *n* the total number of compared taxa *Z* result of sign test (*P* < 0.05)

## Discussion

**Figure 3:**
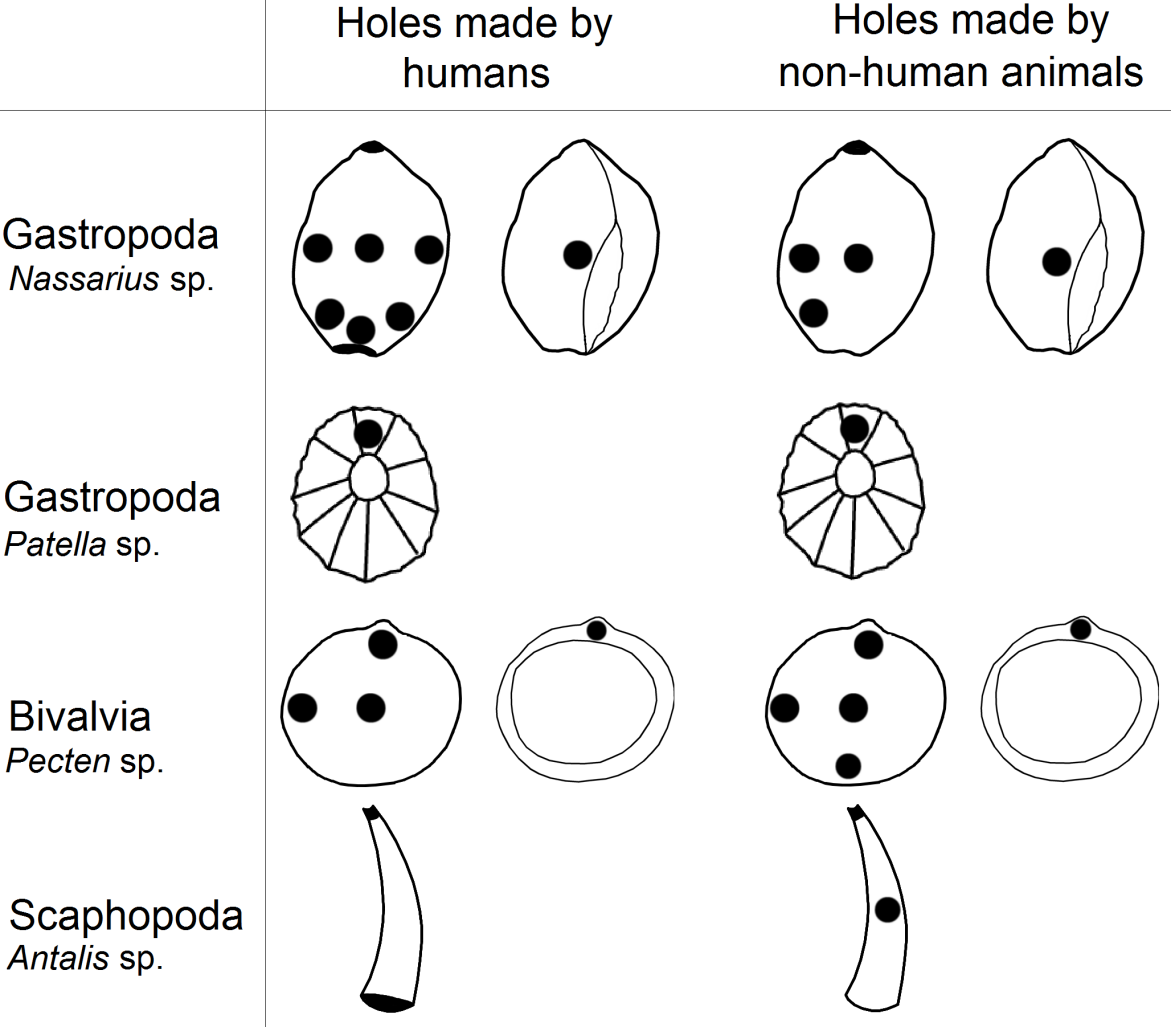
The locations of perforations in shells made by human and non-human animals.

In this study we examined the assertion that the anatomical locations of holes in mollusc shells pierced by prehistoric human groups have lower variability compared to predator made holes, which have been said to be more randomly placed (Stiner, 1999; Bouzouggar et al., 2007). We found that holes in shells reported to have been pierced by humans were as variable as those made by predators. Furthermore, predators and humans pierced shells in similar locations (Fig. 3).

### Holes in the shells of Gastropoda

Among molluscs, species within the Gastropoda were found to be the most varied in terms of hole locations in shell beads made by humans (Fig. 2A, Table 4). This may be associated with natural morphological variation (shape, size and shell thickness) among species in this mollusc class (Stiner, 2014). Different styles of Palaeolithic bead adornments using ornate shells (Stiner, 2014) may also increase the variability of hole placements in the analysed shells.

Almost all gastropod taxa reported from archaeological sites are also vulnerable to hole-boring predators (e.g., Fig. 2B). In most cases, the predators belong to the class Gastropoda or Malacostraca (Table 2). The rest belong to Aves, Asteroidea, Cephalopoda and Polychaeta (Table 2). Despite the numerous predators, gastropod taxa had lower variation in the types of hole location per species made by non-human species than by humans (Table 4). These findings are supported by other research which shows that anatomical locations selected by predators of gastropods are not randomly selected, but are strategically located (Arpad, 1993; Zagyvai & Demeter, 2008). For example, in *Neritina picta*, access to the apex of the shell is preferred as a strategic location by predators belonging to Asteroidea, Muricidae, or Polychaeta (Zagyvai & Demeter, 2008). In turn, *Theodoxus* sp. usually exhibit a muricid (predatory sea snail) borehole that is often located close to the umbilicus (Arpad, 1993).

### Holes in the shells of Scaphopoda

Scaphopoda was the least diverse mollusc class in terms of variation in hole location in shell beads made by humans (Table 4), which is probably a consequence of their characteristic anatomy (tusk shaped). All shell beads belonging to this class have two anatomical holes, one in the anterior (allowing the burrowing foot and captacula to protrude) and a second one in the posterior part of the shell (responsible for respiration; Reynolds, 2002). As such, these shells can be threaded onto a cord without being pierced.

Mean variation of hole location in shells pierced by predators of scaphopods was greater than in shells modified by humans (Table 4). Klompmaker (2011) found that predators pierced holes in the shells of Miocene scaphopods in the middle section of the shell, which is the thickest part. Whereas we found that *Dentalium* sp. was pierced in two places: in the middle or at the posterior part of the shell. However, we were only able to assess predator made holes in one species of Scaphopoda (*Dentalium* sp.), therefore results for this class should be interpreted with caution.

### Holes in the shells of Bivalvia

Holes pierced by humans in bivalves were slightly more diverse than in scaphopods, but were less variable than in gastropods (Table 4). According to Carter (2008) bivalves in South America were rarely used as beads or pendants due to their size and weight and it is possible that most perforations could be attributed to predation or taphonomic processes. Other evidence, based upon context and use-wear analysis of shells from Palaeolithic sites, suggests the presence of bivalves can often be attributed to utilitarian purposes (e.g., food, receptacles for pigments) rather than use as body adornments (Harper, 2005; Bar-Yosef-Mayer, 2007; Rogalla & Amler, 2007; Douka et al., 2014). Zilhão et al. (2010) suggest that for most species of bivalves recovered from archaeological sites, anthropogenic modifications can be confirmed when (i) the weathering stage and perforation patterns do not agree with those seen in natural death assemblages; (ii) a tool was involved in the perforation, or (iii) the hole is associated with artificial modification of the shell’s geometry.

Predators of bivalves are also diverse and belong to Asteroidea, Gastropoda or Malacostraca. This variation in predators might be associated with higher variations in types of hole placement per species, which is slightly greater than reported in anthropogenically modified shells (Table 4). For example, variation in hole location is very low in *Chlamys* sp., with naticid and muricid predators usually choosing the region near the adductor muscle (corresponding to number 2 in Fig. 1), which may facilitate access to the viscera (Chattopadhyay & Dutta, 2013). Similar behaviour to non-human predators has been noted in the fossil bivalve *Pseudodon* in which 33% of holes were made near the anterior adductor muscle by *Homo erectus* at Trinil (Joordens et al., 2015). In this mollusc species, the adductor muscles are placed near the 1 and 6 of the hole location types (Fig. 1). Location of the holes made by *H. erectus* may vary from the data presented in our study because *Pseudodon* shells at Trinil, although engraved, appear to have been perforated in order to open the shells to access the meat rather than to be used as body adornments.

Evidence from the study of bivalve species from the early and middle Pleistocene, indicate that shells were most often pierced by non-human predators close to the umbo or near the centre (Amano, 2006). Only few examples of piercings near the adductor muscle have been described, and these could have been caused by the incomplete drill holes in prey which continue to grow after the attack changing the relative position of the incomplete hole (Chattopadhyay & Dutta, 2013).

### Pierced shells; factors to consider

Our results show that within the analysed sample, there were no significant differences between the placement of holes made by non-human animals and those made by humans; both pierce shells in the same locations in most classes of molluscs (Table 4; Fig. 3). For example in *Euthria cornea*, *Gibbula* sp. and *Mitra corniculata* we observed that humans and non-human predators made holes in the same location (type number 4). Similarly, in *Glycymeris* sp. humans and predators pierced the same part of the shell (types number 4 and 9; Tables 1 and 2). However, for some mollusc species selection of hole location was only partial. For example in *Littorina* sp. we observed that humans and non-human animals made holes in the centre of shell (type number 4) and near apex (type number 8), but this species is also perforated by humans near the outer lip (type number 1; Tables 1 and 2).

The non-random piercing of holes by the mollusc predators is widely reported (e.g., Johannesson & Ekendahl, 2002; Kingsley-Smith, Richardson c A & Seed, 2003; Dietl & Kelley, 2006; Gorzelak et al., 2013) and it can be associated with shell thickness of prey which varies across the body, probably due to differential age of the shell whorls and predatory pressure on snails (Rosin et al., 2013). In molluscs with ornamental shells, up to five times more force can be required to make a hole (Dalziel & Boulding, 2005). Predatory gastropods can spend between three to twenty minutes locating a piercing site on their prey’s shell surface and, once the location is fixed, it may take from several hours to several days to pierce the shell, depending on the thickness (Hagadorn & Boyajian, 1997). The variation in shell thickness within and between mollusc species may be linked to why holes in shells most often occur near the lip and in the centre (e.g., types number 1, 2, 3 and 4; Fig. 1) and why humans and non-human predators choose similar locations to pierce shells of Bivalvia and Gastropoda (Fig. 3) However, results for scaphopods deviated from this pattern, with humans and non-human predators piercing holes in different locations (type 8 and 4 versus type 5, respectively; Fig. 1). Similarity in the choice of hole location made by humans and non-human predators in most species of mollusc could cause researchers to wrongly assign shell perforations as anthropogenically manipulated because they believe predator made holes are less likely to be pierced in suitable locations for threading (D’Errico et al., 2005; Bouzouggar et al., 2007).

Bicho & Haws (2008) have suggested that the larger biomass of molluscs in the Palaeolithic likely meant that mollusc gathering formed part of hunter-gatherers’ regular foraging behaviour in Portugal. Furthermore, predator drill frequency in gastropods and bivalves has been estimated to range between 2.8%–50.0% and 8.6%–34.1%, respectively. In contrast, scaphopods were pierced at a much lower rates (0.9% in *Dentalium* sp.; Taraschewski & Paperna, 1982; Yochelson, Dockery & Wolf, 1983; Hagadorn & Boyajian, 1997; Zagyvai & Demeter, 2008; Sawyer, 2010). It is possible that prehistoric people that regularly foraged for molluscs, were more likely exposed to a greater numbers of naturally perforated shells than we might expect. As such, the likelihood of finding shells with predator-made holes in locations suitable for threading could be higher than researchers believe.

Beads made from shells with holes made by natural processes have been identified at archaeological sites, for example, the perforated bead of *Antalis* sp. from the Early Upper Paleolithic site in El Cuco (Spain; Gutiérrez-Zugasti & Cuenca-Solana, 2013). Some researchers claim that shell beads from the Middle and Lower Palaeolithic could have been perforated by natural processes. For example, Bednarik (2015) proposed that predators and parasitic organisms commonly perforate mollusc shells and that it should be expected that naturally perforated shells were used as beads and pendants. Hahn (1972) emphasized that the signs of human manipulation in shells from Aurignacian sites such as Krems-Hundssteig, Willendorf, Kostienki 1 and Sjuren, are not always present and that some holes could have been made by predators. It is possible that before tools were used to bore holes, finding shells with holes in favourable positions for threading into ornate jewellery may have increased their importance or value. Similarly the natural apertures in fish vertebrae or crinoid discs may have made them attractive as items for threading (e.g., see Mussi, 2002).

Finally we would like to draw attention to the common assumption that predator-made holes are mostly made by chemical processes and tend to be round in outline, while shells pierced by humans have elliptical or irregular outlines (Stiner, 1999; Komšo & Vukosavljević, 2011). We would argue, however, that this statement is probably an overgeneralisation because many predators form holes in their prey which range in shape from nearly perfect circles to ellipsoids (Zagyvai & Demeter, 2008). For example, muricids, naticids and cephalopods use their radula to bore into mollusc shells and they can adapt the size and shape of the pierced hole to the morphology of their prey, as a result, bore holes can differ in shape (Walker & Brett, 2002). Bird beaks can also cause cracks and chips to the shells that imitate stone tool use (Ingolfsson & Estrella, 1978; Harper, 2005; Shumaker, Walkup & Beck, 2011). Thus, detailed analytical methods remain critical to identifying the tell-tale signs of anthropogenic manipulation of shells recovered from archaeological sites.

## Conclusions

Some researchers have argued that holes in shells made by predators vary more than holes made by humans. However, our findings show that the variation in hole location on shell beads recovered from archaeological sites did not significantly differ from the locations of predator-made holes. Our assessment of hole location was based on figures from the published literature only, so it is possible that the true level of variation from beads recovered from archaeological sites might be higher (i.e., in collections not described in the literature).

This study highlights how the placement of holes on the shells made by predators can potentially be similar to human activity. Moreover, the likelihood of finding shells with holes made by predators in locations suitable for threading is probably higher than researchers believe. These findings emphasise the importance of the battery of tests currently used to identify whether piercings in shells are made naturally or are anthropogenic modifications. Dispelling assumptions about human and non-human predator hole placements in shells and providing information on the patterns of predation on molluscs can augment these tests in order to contribute to more realistic scenarios of the social and cultural expressions of prehistoric people.

##  Supplemental Information

10.7717/peerj.2903/supp-1 Figure S1 PRISMA Flow Diagram

10.7717/peerj.2903/supp-2 Supplemental Information 1 PRISMA Checklist

10.7717/peerj.2903/supp-3 Supplemental Information 2 Publications about malacological findings in archaeological contexts

## Additional Information and Declarations

### Data Availability

The following information was supplied regarding data availability: The raw data has been supplied as a Supplemental File.
