# Supplementary figures and images for: A systematic review of animal predation creating pierced shells: implications for the archaeological record of the Old World
Source: PeerJ. 2017 Jan 17;5:e2903. doi: 10.7717/peerj.2903 (PMC5244880; doi:10.7717/peerj.2903)

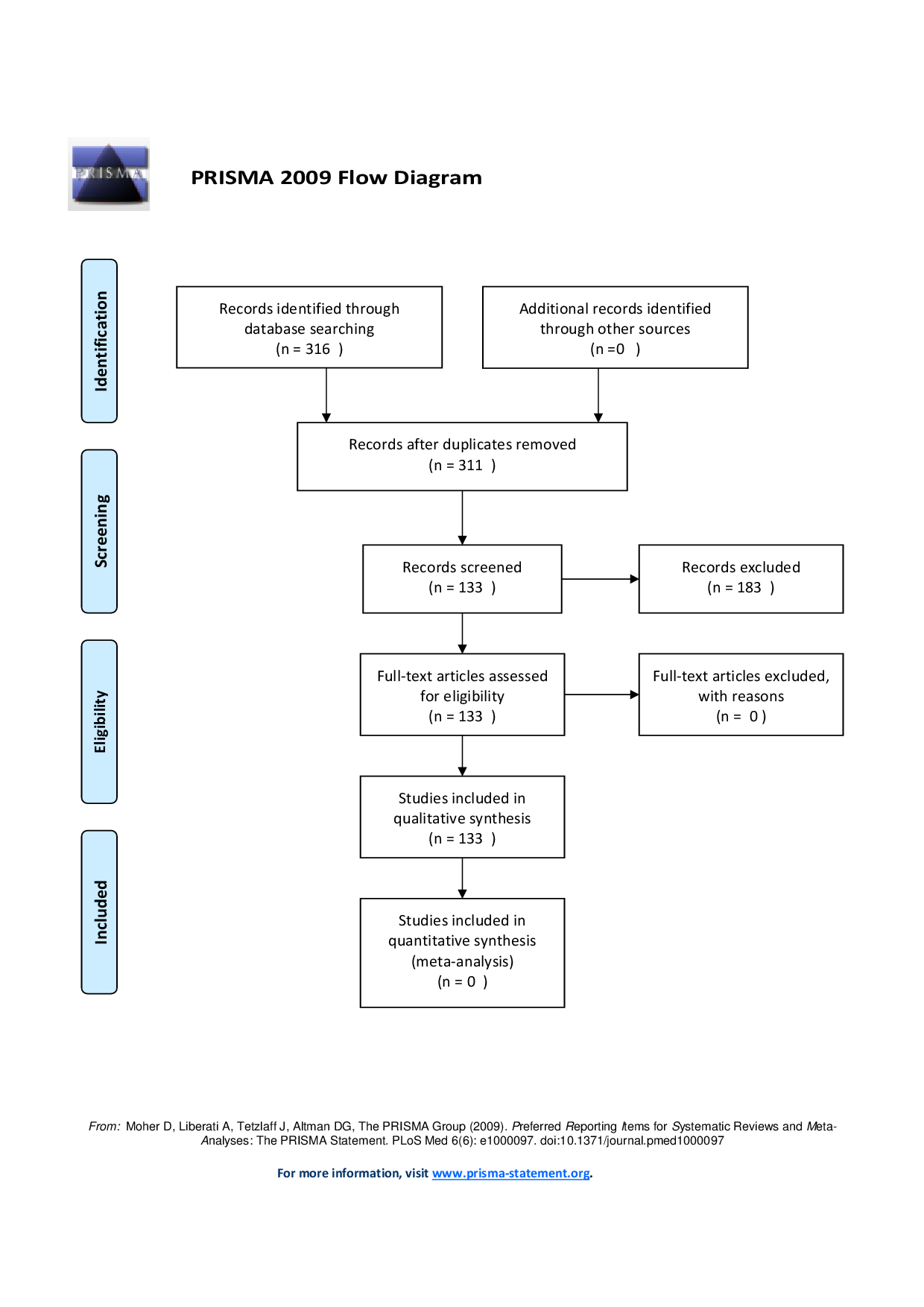

Supplement: Figure S1 [file peerj-05-2903-s001.png]
