# Supplementary material for: A systematic review of animal predation creating pierced shells: implications for the archaeological record of the Old World
Source: PeerJ. 2017 Jan 17;5:e2903. doi: 10.7717/peerj.2903 (PMC5244880; doi:10.7717/peerj.2903)
Supplement: Supplemental Information 2 [file peerj-05-2903-s003.docx]

1. Aiello LC. 1996. Terrestriality, Bipedalism and the Origin of Language. Proceedings of the British Academy 88:269–289.

2. Ainis AF., Vellanoweth RL., Lapeña QG., Thornber CS. 2014. Using non-dietary gastropods in coastal shell middens to infer kelp and seagrass harvesting and paleoenvironmental conditions. Journal of Archaeological Science 49:343–360.

3. Aldeias V., Goldberg P., Dibble HL., El-Hajraoui M. 2014. Deciphering site formation processes through soil micromorphology at Contrebandiers Cave, Morocco. Journal of Human Evolution 69:8–30.

4. Alvarez-Fernandez E., Barrera I., Borja A., Fernandez MJ., Iriarte MJ., Arrizabalaga A. 2013. HOLOCENE Page 2 of 20 Biometric analysis of the stalked barnacle Pollicipes pollicipes (Gmelin, 1790) from a Holocene archaeological site in the Jaizkibel Mountains (Basque Country, Northern Spain). The Holocene 23:1373–1380.

5. Álvarez-Fernández E. 2007. La explotación de los moluscos marinos en la Cornisa Cantábrica durante el Gravetiense: primeros datos de los niveles E Y F De La Garma A (Omoño, Cantabria). Zephyrus 60:43–58.

6. Álvarez-Fernández E. 2009. Magdalenian personal ornaments on the move: A review of the current evidence in Central Europe. Zephyrus 63:45–59.

7. Álvarez-Fernández E. 2010. Shell beads of the Last Hunter-Gatherers and Earliest Farmers in South-Western Europe. Munibe. Antropologia-Arkeologia 61:129–138.

8. Arnold JE. 2012. Detecting Apprentices and Innovators in the Archaeological Record: The Shell Bead-Making Industry of the Channel Islands. Journal of Archaeological Method and Theory 19:269–305.

9. Arnold JE., Munns A. 1994. Independent or Attached Specialization: The Organization of Shell Bead Production in California. Journal of Field Archaeology 21:473–489.

10. Arpad D. 1993. Trace fossils on molluscs from the Molluscan Clay (Late Oligocene, Egerian) - a comparison between two localities (Wind Brickyard, Eger, and Nyárjas Hill Novaj, NE Hungary). Scripta Geologica 2:75–82.

11. Arua I. 1989. Gastropod predators and their dietary preference in an eocene molluscan fauna from Nigeria. Palaeogeography, Palaeoclimatology, Palaeoecology 72:283–290.

12. Aswani S., Flores CF., Broitman BR. 2014. Human harvesting impacts on managed areas: ecological effects of socially-compatible shellfish reserves. Reviews in Fish Biology and Fisheries 25:217–230.

13. Bailey GN., Flemming NC. 2008. Archaeology of the continental shelf: Marine resources, submerged landscapes and underwater archaeology. Quaternary Science Reviews 27:2153–2165.

14. Balme J., Morse K. 2006. Shell beads and social behaviour in Pleistocene Australia. Antiquity 80:799–811.

15. Balter M. 2006. Archaeology. First jewelry? Old shell beads suggest early use of symbols. Science 312:1731.

16. Balter M. 2011. Was North Africa the Launch Pad for Modern Human Migrations? Science 331.

17. Bar-Yosef-Mayer DE. 2010. Opportunities and continuities of shellfish gathering and their relationship to major steps in human evolution : A response to Jerardino. Pyrenae 41:185–189.

18. Bar-Yosef-Mayer DE., Beyin A. 2009. Late Stone Age Shell Middens on the Red Sea Coast of Eritrea. The Journal of Island and Coastal Archaeology 4:108–124.

19. Bar-Yosef Mayer DE. 1997. Neolithic Shell Bead Production in Sinai. Journal of Archaeological Science 24:97–111.

20. Bar-Yosef Mayer DE., Gümüs BA., Islamoglu Y. 2010. Fossil hunting in the neolithic: Shells from the Taurus Mountains at Çatalhöyük, Turkey. Geoarchaeology 25:375–392.

21. Bar-Yosef Mayer DE., Porat N. 2008. Green stone beads at the dawn of agriculture. Proceedings of the National Academy of Sciences of the United States of America 105:8548–8551.

22. Bar-Yosef Mayer DE., Vandermeersch B., Bar-Yosef O. 2009. Shells and ochre in Middle Paleolithic Qafzeh Cave, Israel: indications for modern behavior. Journal of Human Evolution 56:307–314.

23. Barge H. 1983. Essai sur les parures du Paléolithique supérieur dans le sud de la France| la faune malacologique aurignacienne de l’abri Rothschild (Cabrières, Hérault). Bulletin du Musée d’Anthropologie Préhistorique de Monaco Monaco 27:69–83.

24. Barker GM. 2001. The Biology of Terrestrial Molluscs. Oxon: CABI Publishing.

25. Bayman JM. 2002. Hohokam craft economies and the materialization of power. Journal of Archaeological Method and Theory 9:69–95.

26. Baysal E. 2013. A tale of two assemblages: Early Neolithic manufacture and use of beads in the Konya plain. Anatolian Studies 63:1–15.

27. Bednarik RG. 2001. Beads and Pendants of the Pleistocene. Anthropos 96:545–555.

28. Bednarik RG. 2006. Lecture No. 4. Beads, symbolism and self-awareness. In: Semiotix Course 2006, Cognition and symbolism in human evolution. 1–9.

29. Bednarik RG. 2008. Beads and cognitive evolution. Time and Mind: The journal of archaeology, consciousness and culture 1:285–318.

30. Bednarik RG. 2015. The Significance of the Earliest Beads. Advances in Anthropology 5:51–66.

31. Belfer‐Cohen A., Hovers E. 2010. Modernity, Enhanced Working Memory, and the Middle to Upper Paleolithic Record in the Levant. Current Anthropology 51:S167–S175.

32. Benazzi S., Douka K., Fornai C., Bauer CC., Kullmer O., Svoboda J., Pap I., Mallegni F., Bayle P., Coquerelle M., Condemi S., Ronchitelli A., Harvati K., Weber GW. 2011. Early dispersal of modern humans in Europe and implications for Neanderthal behaviour. Nature 479:525–528.

33. Bertola S., Broglio A., Cristiani E., De Stefani M., Gurioli F., Negrino F., Romandini M., Vanhaeren M. 2013. La diffusione del primo Aurignaziano a sud dell’arco alpino. Preistoria Alpina 47:123–152.

34. Beyin A. 2010. Use-wear analysis of obsidian artifacts from Later Stone Age shell midden sites on the Red Sea Coast of Eritrea, with experimental results. Journal of Archaeological Science 37:1543–1556.

35. Bicho N., Haws J. 2008. At the land’s end: Marine resources and the importance of fluctuations in the coastline in the prehistoric hunter-gatherer economy of Portugal. Quaternary Science Reviews 27:2166–2175.

36. Bird DW., Bliege Bird R., Codding BF. 2009. In pursuit of mobile prey: martu hunting strategies and archaeofaunal interpretation. American Antiquity 74:3–29.

37. Blake JA. 1969. Systematics and Ecology of Shell-Boring Polychaetes from New England. American Zoologist 9:813–820.

38. Blakeslee AMH., Byers JE. 2008. Using parasites to inform ecological history: Comparisons among three congeneric marine snails. Ecology 89:1068–1078.

39. Blanco-Libreros JF., Arroyave-Rincón A. 2009. Predator damage and shell size on the diadromous snail Neritina virginea (Gastropoda: Neritidae) in the Mameyes River, Puerto Rico. Revista de biología tropical 57:1069–80.

40. Board A. 2008. 14 C Dating of the Upper Paleolithic Site At Krems-Hundssteig in. Sciences-New York 50.

41. Bogitsh BJ., Carter CE., Oeltmann TN. 1962. Human Parasitology. Waltham: Elsevier.

42. Botha R. 2008. Prehistoric shell beads as a window on language evolution. Language and Communication 28:197–212.

43. Botha R. 2010. On the Soundness of Inferring Modern Language from Symbolic Behaviour. Cambridge Archaeological Journal 20:345–356.

44. Bouzouggar A., Barton N., Vanhaeren M., Collcutt S., Higham T., Hodge E., Par S., Rhodes E., Schwenninger J., Stringer C., Turner E., Ward S., Moutmir A., Stambouli A. 2007. 82,000-year-old shell beads from North Africa and implications for the origins of modern human behavior. Pnas 104:1–6.

45. Bower JRF. 2015. On “Modern Behavior” and the Evolution of Human Intelligence. Current Anthropology 46:121–122.

46. Braje TJ., Rick TC., Reeder-myers L., Campbell B., Minas K. 2014. Defining the historic landscape on eastern Santa Rosa island: archaeological investigations at Qshiwshiw. Monographs of the Western North American Naturalist 7:135–145.

47. Brumm A., Moore MW. 2005. Symbolic Revolutions and the Australian Archaeological Record. Cambridge Archaeological Journal 15:157–175.

48. Cáceres M., Marcos G., Diez G. 2008. Upper Paleolithic ornament seashell from Sala de las Chimeneas , Maltravieso cave ( Cáceres , Spain ). In: Álvarez-Fernández E, Carcajal-Contreras D eds. Not only food. Marine, Terrestrial and Freshwater Molluscs in Archaeological Sites. Proceedings of the 2nd Meeting of the ICAZ Archaeomalacology Working Group (Santander, February 19th – 22nd 2008). Aranzadi Zientzia Elkartea, 19–22.

49. Calvet C. 1992. Borehole site-selection in Naticarius hebraeus (Chemnitz in Karsten, 1769) (Naticidae : Gastropoda)? Orsis 7:57–64.

50. Carannante A. 2011. Purple-Dye Industry shell waste Recycling in the Bronze Age Aegean? Stoves and Murex shells at Minoan Monastiraki (Crete, Greece). In: Çakırlar C ed. Archaeomalacology revisited. Non-dietary use of molluscs in Archaeological settings. Oxbow Books, 9–18.

51. Cardoso JL., Dias Coelho M. 2012. The marine malacological remains from the chalcolithic fortified settlement at Outeiro Redondo (Sesimbra): Collection strategies used by a sedentary community from the 3rd millennium BC on the portuguese coast. Zephyrus 70:85–111.

52. Carter BP. 2008. Technology, Society and Change: Shell Artifact Production Among the Manteno (A.D. 800--1532) of Coastal Ecuador. ProQuest.

53. Charalambidou I., Santamaria L. 2002. Water birds as endozoochorous dispersers of aquatic organisms: a review of experimental evidence. Acta Oecologica 23:165–176.

54. Chattopadhyay D., Dutta S. 2013. Prey selection by drilling predators: A case study from Miocene of Kutch, India. Palaeogeography, Palaeoclimatology, Palaeoecology 374:187–196.

55. Christensen CC., Kirch V. 1981. Nonmarine Molluscs from Archaeological Sites on Tikopia, Southeastern Solomon Islands. Pacific Science 35:75–88.

56. Claassen C. 2011. Shell Symbolism in Pre-Columbian North America. In: Antczak A, Cipriani R eds. Early Human Impact on Megamolluscs. Oxford: British Archeological Reports, 37–43.

57. Coleman HM. 2010. Complex species interaction in tropical backreef communities. Journal of Experimental Marine Biology and Ecology 393:124–129.

58. Conard NJ. 2010. Cultural modernity: consensus or conundrum? Proceedings of the National Academy of Sciences of the United States of America 107:7621–7622.

59. Contreras DRC. 2010. Shell Artefacts from the Gold Museum in Colombia: A View from the Intermediate Area. In: Çakırlar C ed. Archaeomalacology Revisited Non-dietary use of Molluscs in Archaeological Settings. Oxbow Books, 19–29.

60. Cook LM., Kenyon G. 1993. Shell strength of colour morphs of the mangrove snail Littoraria pallescens. Journal of Molluscan Studies 59:29–34.

61. Corchón MS., Ortega P., González D., Muñoz Á., Rodríguez P., Gárate D., Rivero O. 2012. New research studies in the cave of La Griega (Pedraza, Segovia, Spain). Contribution of geotechnologies to the study of palaeolithic art. Espacio, Tiempo y Forma. Serie I, Prehisoria y Arqueología. 5:543–556.

62. Coupe C. 2010. Semiotic investigation into early forms of symbolism and language. In: The Evolution of Language (Proceedings of the 9th Evolang Conference). 1–8.

63. Creamer W., Haas J., Jakaitis E., Holguin J. 2011. Far From the Shore: Comparison of Marine Invertebrates in Midden Deposits From Two Sites in the Norte Chico, Peru. The Journal of Island and Coastal Archaeology 6:176–195.

64. Cristiani E. 2012. Ornamental Traditions of the Late Pleistocene and the Early Holocene Foragers in the Eastern Alps: the Case of Riparo Biarzo. Geologia, Paleontologia, Paletnologia 34:89–102.

65. Cristiani E., Živaljević I., Borić D. 2014. Residue analysis and ornament suspension techniques in prehistory: Cyprinid pharyngeal teeth beads from Late Mesolithic burials at Vlasac (Serbia). Journal of Archaeological Science 46:292–310.

66. Cruz S., Rosa S. 2015. Shell Beads as Indicators of Wealth and Rank. In: Gamble LH ed. The Chumash World at European Contact : Power, Trade, and Feasting Among Complex Hunter-gatherers. California: University of California Press, 229–239.

67. Cuenca-Solana D., Gutiérrez-Zugasti FI., González-Morales MR., Setién-Marquinez J., Ruiz-Martinez E., García-Moreno A., Clemente-Conte I. 2013. Shell Technology, Rock Art, and the Role of Marine Resources during the Upper Paleolithic. Current Anthropology 54:370–380.

68. D’Anastasio R., Wroe S., Tuniz C., Mancini L., Cesana DT., Dreossi D., Ravichandiran M., Attard M., Parr WCH., Agur A., Capasso L. 2013. Micro-biomechanics of the Kebara 2 hyoid and its implications for speech in Neanderthals. PLoS ONE 8.

69. d’Errico F., Henshilwood C., Vanhaeren M., van Niekerk K. 2005. Nassarius kraussianus shell beads from Blombos Cave: Evidence for symbolic behaviour in the Middle Stone Age. Journal of Human Evolution 48:3–24.

70. d’Errico F., Vanhaeren M., Barton N., Bouzouggar A., Mienis H., Richter D., Hublin J-J., McPherron SP., Lozouet P. 2009. Out of Africa: modern human origins special feature: additional evidence on the use of personal ornaments in the Middle Paleolithic of North Africa. Proceedings of the National Academy of Sciences of the United States of America 106:16051–16056.

71. d’Errico F., Salomon H., Vignaud C., Stringer C. 2010. Pigments from the Middle Palaeolithic levels of Es-Skhul (Mount Carmel, Israel). Journal of Archaeological Science 37:3099–3110.

72. d’Errico F., Backwell L., Villa P., Degano I., Lucejko JJ., Bamford MK., Higham TFG., Colombini MP., Beaumont PB. 2012. Early evidence of San material culture represented by organic artifacts from Border Cave, South Africa. Proceedings of the National Academy of Sciences 109:13214–13219.

73. D’Errico F. 2003. The Invisible Frontier. A Multiple Species Model for the Origin of Behavioral Modernity. Evolutionary Anthropology 12:188–202.

74. D’Errico F., Vanhaeren M., Barton N., Bouzouggar A., Mienis H., Richter D., Hublin J-J., McPherron SP., Lozouet P. 2009. Additional evidence on the use of personal ornaments in the Middle Paleolithic of North Africa. Proceedings of the National Academy of Sciences 106:18040.

75. d’Errico F., Stringer CB. 2011. Evolution, revolution or saltation scenario for the emergence of modern cultures? Philosophical Transactions of the Royal Society B: Biological Sciences 366:1060–1069.

76. d’Errico F., Vanhaeren M. 2009. Additional evidence on the use of personal ornaments in the Middle Paleolithic of North Africa. Proceedings of the National Academy of Sciences of the United States of America 106:16051–16056.

77. d’Errico F., Vanhaeren M., Wadley L. 2008. Possible shell beads from the Middle Stone Age layers of Sibudu Cave, South Africa. Journal of Archaeological Science 35:2675–2685.

78. Dalziel B., Boulding EG. 2005. Water-borne cues from a shell-crushing predator induce a more massive shell in experimental populations of an intertidal snail. Journal of Experimental Marine Biology and Ecology 317:25–35.

79. David B., Aplin K., Petchey F., Skelly R., Mialanes J., Jones-Amin H., Stanisic J., Barker B., Lamb L. 2015. Kumukumu 1, a hilltop site in the Aird Hills: Implications for occupational trends and dynamics in the Kikori River delta, south coast of Papua New Guinea. Quaternary International 385:7–26.

80. Day EG., Branch GM., Viljoen C. 2000. How costly is molluscan shell erosion? A comparison of two patellid limpets with contrasting shell structures. Journal of Experimental Marine Biology and Ecology 243:185–208.

81. Delance JH., Emig CC. 2004. Drilling predation on Gryphus vitreus (Brachiopoda) off the French Mediterranean coasts. Palaeogeography, Palaeoclimatology, Palaeoecology 208:23–30.

82. Deline B. 2008. The First Evidence of Predatory or Parasitic Drilling in Stylophoran Echinoderms. Acta Palaeontologica Polonica 53:739–743.

83. Deshpande-Mukherjee A. 2005. Marine shell utilisation by the Chalcolithic societies of the western Deccan region of India. In: Bar-Yosef-Mayer D ed. Archaeomalacology. Molluscs in former environments of human behaviour. Oxford: Oxbow Books, 174–184.

84. Dodge R., Scheel D. 1999. Remains of the prey - Recognizing the midden piles of Octopus dofleini (Wülker). The Veliger 42:260–266.

85. Douka K. 2011. An Upper Palaeolithic shell scraper from Ksar Akil (Lebanon). Journal of Archaeological Science 38:429–437.

86. Douka K., Bergman CA., Hedges REM., Wesselingh FP., Higham TFG. 2013. Chronology of Ksar Akil (Lebanon) and implications for the colonization of Europe by anatomically modern humans. PloS one 8:e72931.

87. Douka K. 2013. The Chronology of the Middle to the Upper Paleolithic Transition in the Northern Levant. Mitteilungen der Gesellschaft Urgeschichte 22:11–40.

88. Douka K., Higham TFG., Wood R., Boscato P., Gambassini P., Karkanas P., Peresani M., Ronchitelli AM. 2014. On the chronology of the Uluzzian. Journal of Human Evolution 68:1–13.

89. Duarte CM. 2014. Red ochre and shells: clues to human evolution. Trends in Ecology & Evolution 29:560–565.

90. Eastham A. 1997. The potential of bird remains for environmental reconstruction. International Journal of Osteoarchaeology 7:422–429.

91. Edgell T., Brazeau C., Grahame J., Rochette R. 2008. Simultaneous defense against shell entry and shell crushing in a snail faced with the predatory shorecrab Carcinus maenas. Marine Ecology Progress Series 371:191–198.

92. Edgell TC., Rochette R. 2009. Prey-induced changes to a predator’s behaviour and morphology: Implications for shell–claw covariance in the northwest Atlantic. Journal of Experimental Marine Biology and Ecology 382:1–7.

93. Eilertsen MH., Malaquias MAE. 2013. Unique digestive system, trophic specialization, and diversification in the deep-sea gastropod genus Scaphander. Biological Journal of the Linnean Society 109:512–525.

94. Erlandson JM., Macko ME., Koerper HC., Southon J. 2005a. The antiquity of Olivella shell beads at CA-ORA-64: AMS radiocarbon dated between 9420 and 7780 cal BP. Journal of Archaeological Science 32:393–398.

95. Erlandson JM., Braje TJ., Rick TC., Peterson J. 2005b. Beads, Bifaces, and Boats: An Early Maritime Adaptation on the South Coast of San Miguel Island, California. American Anthropologist 107:677–683.

96. Erlandson JM., Moss ML., Des Lauriers M. 2008. Life on the edge: early maritime cultures of the Pacific Coast of North America. Quaternary Science Reviews 27:2232–2245.

97. Estrada A., Tejero JM., Mangado X., Petit MA., Fullola JM., Esteve X., Bartroli R. 2010. From the Mediterranean sea to the Segre river: manipulated shells f1. Estrada A, Tejero JM, Mangado X, et al. (2010) From the Mediterranean sea to the Segre river: manipulated shells from Magdalenian levels of Parco’s cave (Alòs de Balaguer, Lleida Spain). In: Álvarez-Fernández E, Carcajal-Contreras D eds. Not only food. Marine, Terrestrial and Freshwater Molluscs in Archaeological Sites. Proceedings of the 2nd Meeting of the ICAZ Archaeomalacology Working Group (Santander, February 19th – 22nd 2008). Donostia: Aranzadi Zientzia Elkartea, 70–77.

98. Faith JT. 2013. Taphonomic and paleoecological change in the large mammal sequence from Boomplaas Cave, western Cape, South Africa. Journal of Human Evolution 65:715–730.

99. Fano MÁ., Álvarez-Fernández E. 2010. Magdalenian marine shells from El Horno Cave (Ramales , Cantabria , Spain) in the regional context. In: Alvarez-Fernandez E, Carvajal-Contreras DR eds. Not only food. Marine, Terrestrial and Freshwater Molluscs in Archaeological Sites. Proceedings of the 2nd Meeting of the ICAZ Archaeomalacology Working Group (Santander, February 19th – 22nd 2008). Santander: Munibe, 58–68.

100. Fernández-López de Pablo J., Badal E., Ferrer García C., Martínez-Ortí A., Sanchis Serra A. 2014. Land Snails as a Diet Diversification Proxy during the Early Upper Palaeolithic in Europe. PloS one 9:e104898.

101. Filipescu S., Popa M. 2001. Biostratigraphic and paleoecologic significance of the macro- and microfossil assemblages in the Borod Formation (eastern Borod Depression, north-west Romania). Acta Palaeontologica Romaniae 3:135–148.

102. Fontana F., Cilli C., Cremona MG., Giacobini G., Guiriolo F., Liagre J., Malerba G., Rocci Ris A., Veronese C., Guerreschi A. 2009. Recent data on the Late Epigravettian occupation at Riparo Tagliente, Monti Lessini (Grezzana, Verona): a multidisciplinary perspective. Preistoria Alpina 44:49–57.

103. Francis P. 1989. The manufacture of beads from shell. In: Proceedings of the 1986 Shell Bead Conference: selected papers. Rochester Museum and Science Center. Research Division, 25–35.

104. García-Aguilar JM., Guerra-Merchán A., Serrano F., Palmqvist P., Flores-Moya A., Martínez-Navarro B. 2014. Hydrothermal activity and its paleoecological implications in the latest Miocene to Middle Pleistocene lacustrine environments of the Baza Basin (Betic Cordillera, SE Spain). Quaternary Science Reviews 96:204–221.

105. Gliganic L a., Jacobs Z., Roberts RG., Domínguez-Rodrigo M., Mabulla AZP. 2012. New ages for Middle and Later Stone Age deposits at Mumba rockshelter, Tanzania: Optically stimulated luminescence dating of quartz and feldspar grains. Journal of Human Evolution 62:533–547.

106. Godfrey-Smith P. 2014. Signs and Symbolic Behavior. Biological Theory 9:78–88.

107. Goebel T. 2007. Anthropology. The missing years for modern humans. Science 315:194–196.

108. Golovanova L V., Doronichev VB., Cleghorn NE. 2010. The emergence of bone-working and ornamental art in the Caucasian Upper Paleolithic. Antiquity 84:299–320.

109. González-Solis J., Abella JC., Aymi R. 1996. Shell size relationships in the consumption of gastropods by migrant Song Thrushes Turdus philomelos. October 20:147–149.

110. Gordillo S. 2013. Cannibalism in Holocene muricid snails in the Beagle Channel, at the extreme southern tip of South America : an opportunistic response? Palaeontologia Electronica 16:1–13.

111. Gordillo S., Archuby F. 2014. Live-live and live-dead interactions in marine death assemblages: the case of the Patagonian clam Venus antiqua. Acta Palaeontologica Polonica 59:429–442.

112. Gorman D., Sikinger CE., Turra A. 2015. Spatial and temporal variation in the predation risk for hermit crabs in a subtropical bay. Journal of Experimental Marine Biology and Ecology 462:98–104.

113. Gorzelak P., Salamon MA., Trzęsiok D., Niedźwiedzki R. 2013. Drill holes and predation traces versus abrasion-induced artifacts revealed by tumbling experiments. PloS one 8:e58528.

114. Grey M. 2005. Shape differences among boreholes drilled by three species of Naticid Gastropods. Journal of Molluscan Studies 71:253–256.

115. Grey M., Lelievre PG., Boulding EG. 2005. Selection for Prey Shell Thickness by the Naticid Gastropod Euspira lewisii (Naticidae) on the Bivalve Protothaca staminea (Veneridae). The Veliger 48:6–11.

116. Guerra A., Nixon M. 1987. Crab and mollusc shell drilling by Octopus vulgaris (Mollusca: Cephalopoda) in the Ria de Vigo (north-west Spain). Journal of Zoology 211:515–523.

117. Guerrero Alba S., Reyment R a. 1988. Differentiation Between the Traces of Predation of Muricids and Naticids in Spanish Pliocene Chlamys. Estudios geol. 44:317–328.

118. Gutiérrez-Zugasti I., Cuenca-Solana D., Rasines del Río P., Muñoz E., Santamaría S., Morlote JM. 2013. The role of shellfish in hunter-gatherer societies during the Early Upper Palaeolithic: A view from El Cuco rockshelter, northern Spain. Journal of Anthropological Archaeology 32:242–256.

119. Haddad V., Neto JBDP., Cobo VJ. 2006. Venomous mollusks: The risks of human accidents by Conus snails (Gastropoda: Conidae) in Brazil. Revista da Sociedade Brasileira de Medicina Tropical 39:498–500.

120. Haidle MN., Pawlik AF. 2011. Pleistocene Modernity: An Exclusively Afro-European Issue? An Introduction to Session A1. Bulletin of the Indo-Pacific Prehistory Association 30:3–8.

121. Harding JM., Kingsley-Smith P., Savini D., Mann R. 2007. Comparison of predation signatures left by Atlantic oyster drills (Urosalpinx cinerea Say, Muricidae) and veined rapa whelks (Rapana venosa Valenciennes, Muricidae) in bivalve prey. Journal of Experimental Marine Biology and Ecology 352:1–11.

122. Hardy K. 2008. Prehistoric string theory. How twisted fibres helped to shape the world. Antiquity 82:271–280.

123. Harper EM., Robinson JH., Lee DE. 2011. Drill hole analysis reveals evidence of targeted predation on modern brachiopods. Palaeogeography, Palaeoclimatology, Palaeoecology 305:162–171.

124. Harzhauser M., Lenneis E., Neugebauer-Maresch C. 2007. Freshwater gastropods as Neolithic adornment: size selectiveness and perforation morphology as a result of grinding techniques. Anthropologie und Prahistoire 109:73–85.

125. Hendricks JR. 2015. Glowing Seashells: Diversity of Fossilized Coloration Patterns on Coral Reef-Associated Cone Snail (Gastropoda: Conidae) Shells from the Neogene of the Dominican Republic. PLoS ONE:1–59.

126. Henry-Gambier D. 2002. Les fossiles de Cro-Magnon (Les Eyzies-de-Tayac, Dordogne). Bulletins et memoires de la Societe d’Anthropologie de Paris 14:89–112.

127. Henshilwood C., D’Errico F., Vanhaeren M., van Niekerk K., Jacobs Z. 2004. Middle Stone Age shell beads from South Africa. Science 304:404.

128. Henshilwood CS. 2012. Late Pleistocene Techno-traditions in Southern Africa: A Review of the Still Bay and Howiesons Poort, c. 75-59 ka. Journal of World Prehistory 25:205–237.

129. Henshilwood CS., Dubreuil B. 2011. The Still Bay and Howiesons Poort, 77–59 ka. Current Anthropology 52:361–400.

130. Higham CFW., Guangmao X., Qiang L. 2011. The prehistory of a Friction Zone: first farmers and hunters-gatherers in Southeast Asia. Antiquity 85:529–543.

131. Hladilová Š., Fordinál K. 2013. Upper Badenian Molluscs (Gastropoda, Bivalvia, Scaphopoda) from the Modra-Kráľová locality (Danube Basin, Slovakia). 45:35–44.

132. Hodgson D. 2014. Decoding the Blombos Engravings, Shell Beads and Diepkloof Ostrich Eggshell Patterns. Cambridge Archaeological Journal 24:57–69.

133. Holden C. 2004. Oldest Beads Suggest Early Symbolic Behavior. Science 304.

134. Hublin J-J. 2012. The earliest modern human colonization of Europe. Proceedings of the National Academy of Sciences 109:13471–13472.

135. Huntley JW., Baets K De. 2016. Trace Fossil Evidence of Trematode e Bivalve Parasite e Host Interactions in Deep Time. Advances in Parasitology 90:1–31.

136. Ingolfsson A., Estrella B. 1978. The Development of Shell-cracking Behavior in Herring Gulls. Auk 95:577–579.

137. Inizan M-L. 1978. Coquillages de Ksar-’Aqil: éléments de parure? Paléorient 4:295–306.

138. Ishikawa M., Kase T. 2007. Multiple predatory drill holes in Cardiolucina (Bivalvia: Lucinidae): Effect of conchiolin sheets in predation. Palaeogeography, Palaeoclimatology, Palaeoecology 254:508–522.

139. Jacobs Z., Duller G a T., Wintle AG., Henshilwood CS. 2006. Extending the chronology of deposits at Blombos Cave, South Africa, back to 140 ka using optical dating of single and multiple grains of quartz. Journal of Human Evolution 51:255–273.

140. Jacobs Z., Roberts RG., Galbraith RF., Deacon HJ., Grün R., Mackay A., Mitchell P., Vogelsang R., Wadley L. 2008. Ages for the Middle Stone Age of Southern Africa: Implications for Human Behavior and Dispersal. Science 322.

141. Jacobs Z., Meyer MC., Roberts RG., Aldeias V., Dibble H., El Hajraoui M a. 2011. Single-grain OSL dating at La Grotte des Contrebandiers (’Smugglers’ Cave’), Morocco: Improved age constraints for the Middle Paleolithic levels. Journal of Archaeological Science 38:3631–3643.

142. Jacobs Z., Hayes EH., Roberts RG., Galbraith RF., Henshilwood CS. 2013. An improved OSL chronology for the Still Bay layers at Blombos Cave, South Africa: Further tests of single-grain dating procedures and a re-evaluation of the timing of the Still Bay industry across southern Africa. Journal of Archaeological Science 40:579–594.

143. Jacobs Z., Roberts RG. 2009. Catalysts for Stone Age innovations. Communicative & Integrative Biology Publication 2:191–193.

144. Janetski JC., Bar-Yosef ED. 2005. Shifts in Epipaleolithic marine shell exploitation at wadi Mataha, southern Jordan. In: Bar-Yosef Mayer D ed. Archaeomalacology. Molluscs in former environments of human behaviour. Oxford: Oxbow Books, 149–158.

145. Jazwa CS., Gamble LH., Kennett DJ. 2013. A high-precision chronology for two jouse features at an early village site on western Santa Cruz Island, California, Usa. Radiocarbon 55:185–199.

146. Jazwa CS., Mather R. 2014. Archaeological Site or Natural Marine Community? Excavation of a Submerged Shell Mound in Ninigret Pond, Rhode Island. The Journal of Island and Coastal Archaeology 9:268–288.

147. Jenkins DL., Erlandson JM. 1996. Olivella Grooved Rectangle Beads from a Middle Holocene Site in the Fort Rock Valley, Northern Great Basin on JSTOR. Journal of California and Great Basin Anthropology 18:296–302.

148. Jerardino A. 2012. Large Shell Middens and Hunter-Gatherer Resource Intensification Along the West Coast of South Africa: The Elands Bay Case Study. The Journal of Island and Coastal Archaeology 7:76–101.

149. Jerardino A. 2014a. Variability in late Holocene shellfish assemblages: the significance of large shore barnacles (Austromegabalanus cylindricus) in South African West Coast sites. Journal of Archaeological Science 52:56–63.

150. Jerardino A. 2014b. Stranded rocky shore mussels and their possible procurement during prehistory on the West Coast of South Africa. Journal of Archaeological Science 49:536–545.

151. Jerardino A., Marean CW. 2010. Shellfish gathering, marine paleoecology and modern human behavior: Perspectives from cave PP13B, Pinnacle Point, South Africa. Journal of Human Evolution 59:412–424.

152. Johannesson K., Ekendahl A. 2002. Selective predation favouring cryptic individuals of marine snails (Littorina). Biological Journal of the Linnean Society 76:137–144.

153. Jonkers HA. 2000. Gastropod predation patterns in Pliocene and Recent pectinid bivalves from Antarctica and New Zealand. New Zeland Journal of Geology & Geophysics 43:247–254.

154. Joordens J., D’Errico F., Wesselingh F., Munro S., de Vos J., Wallinga J., Ankjærgaard C., Reimann T., Wijbrans J., Kuiper K., Mücher H., Coqueugniot H., Prié V., Joosten I., van Os B., Schulp A., Panuel M., van der Haas V., Lustenhouwer W., Reijmer J., Roebroeks W. 2015. Homo erectus at Trinil on Java used shells for tool production and engraving. Nature 518:228–231.

155. Jordaens K., De Wolf H., Vandecasteele B., Blust R., Backeljau T. 2006. Associations between shell strength, shell morphology and heavy metals in the land snail Cepaea nemoralis (Gastropoda, Helicidae). Science of the Total Environment 363:285–293.

156. Juan-Foucher C., Foucher P. 2008. Marine shell beads from the Gravettian at Gargas cave (Hautes- Pyrenees, France): cultural and territorial markers. In: Álvarez-Fernández E, Carcajal-Contreras D eds. Not only food. Marine, Terrestrial and Freshwater Molluscs in Archaeological Sites. Proceedings of the 2nd Meeting of the ICAZ Archaeomalacology Working Group (Santander, February 19th – 22nd 2008). Donostia: Aranzadi Zientzia Elkartea, 28–35.

157. Karklins K. 2015. Researching the world’s beads: an annotated bibliography.

158. Kawakami K., Wada S., Chiba S. 2008. Possible dispersal of land snails by birds. Ornithological Science 7:167–171.

159. Kelley JT., Belknap DF., Kelley AR., Claesson SH. 2013. A model for drowned terrestrial habitats with associated archeological remains in the northwestern Gulf of Maine, USA. Marine Geology 338:1–16.

160. Kherbouche F., Hachi S., Abdessadok S., Sehil N., Merzoug S., Sari L., Benchernine R., Chelli R., Fontugne M., Barbaza M., Roubet C. 2014. Preliminary results from excavations at Gueldaman Cave GLD1 (Akbou, Algeria). Quaternary International 320:109–124.

161. Kiatkamjornwong S., Akkarakittimongkol P., Omi S. 2002. Syntheses of acrylate core/shell imbiber beads by seeded suspension copolymerization and one-stage copolymerization for solvent absorption-desorption. Journal of Applied Polymer Science 85:670–682.

162. Kidd KE. 1953. The Excavation and Historical Identification of a Huron Ossuary. American Antiquity 18:359.

163. Kidwell SM., Aigner T. 1985. Sedimentary dynamics of complex shell beds: implications for ecologic and evolutionary patterns. In: Bayer U, Seilacher A eds. Sedimentary and Evolutionary Cycles. Springer Berlin Heidelberg, 382–395.

164. Kingsley-Smith PPR., Richardson C a. C., Seed R. 2003. Stereotypic and size-selective predation in Polinices pulchellus (Gastropoda: Naticidae) Risso 1826. Journal of Experimental Marine Biology and Ecology 295:173–190.

165. Kitching J a., Lockwood J. 1974. Observations on shell form and its ecological significance in thaisid gastropods of the genus Lepsiella in New Zealand. Marine Biology 28:131–144.

166. Kleewein D. 1999. Population size, density, spatial distribution and dispersal in an Austrian population of the land snail Arianta arbustorum styriaca (Gastropoda: Helicidae). Journal of Molluscan Studies 65:303–315.

167. Klein RG., Steele TE. 2013. Archaeological shellfish size and later human evolution in Africa. Proceedings of the National Academy of Sciences of the United States of America 110:10910–10915.

168. Klingenberg CP. 2015. Analyzing fluctuating asymmetry with geometric morphometrics: Concepts, methods, and applications. Symmetry 7:843–934.

169. Klompmaker AA. 2011. Drilling and crushing predation on scaphopods from the Miocene of the Netherlands. Lethaia 44:429–439.

170. Kohn AJ., Arua I. 1999. An early pleistocene molluscan assemblage from Fiji: Gastropod faunal composition, paleoecology and biogeography. Palaeogeography, Palaeoclimatology, Palaeoecology 146:99–145.

171. Komšo D., Vukosavljević N. 2011. Connecting coast and inland: Perforated marine and freshwater snail shells in the Croatian Mesolithic. Quaternary International 244:117–125.

172. Kowalewski M. 2004. Drill holes produced by the predatory gastropod Nucella Lamellosa (Muricidae): Palaebiological and ecological implications. Journal Molluscan Studies 70:359–370.

173. Kowalewski M., Dulai A., Fürsich FT. 1998. A fossil record full of holes: The Phanerozoic history of drilling predation. Geology 26:1091–1094.

174. Kozuch L. 2002. Olivella Beads from Spiro and the Plains. American Antiquity 67:697.

175. Kuhn SL., Stiner MC., Reese DS., Güleç E. 2001. Ornaments of the earliest Upper Paleolithic: Proceedings of the National Academy of Sciences of the United States of America 98:7641–7646.

176. Kuhn SL. 2002. Paleolithic Archeology in Turkey. Evolutionary Anthropology 11:198–210.

177. Kuhn SL. 2014. Signaling Theory and Technologies of Communication in the Paleolithic. Biological Theory 9:42–50.

178. Kuhn SL., Stiner MC. 1998. The Earliest Aurignacian of Riparo Mochi (Liguria, Italy). Current Anthropology 39:s175–s189.

179. Kyriacou K., Parkington JE., Will M., Kandel A., Conard NJ. 2015. Middle and Later Stone Age shellfish exploitation strategies and coastal foraging at Hoedjiespunt and Lynch Point, Saldanha Bay, South Africa. Journal of Archaeological Science 57:197–206.

180. Larson LH. 1971. Archaeological Implications of Social Stratification at the Etowah Site, Georgia on JSTOR. Memoirs of the Society for American Archaeology 25:58–67.

181. Latorre C., Santoro CM., Ugalde PC., Gayo EM., Osorio D., Salas-Egaña C., De Pol-Holz R., Joly D., Rech J a. 2013. Late Pleistocene human occupation of the hyperarid core in the Atacama Desert, northern Chile. Quaternary Science Reviews 77:19–30.

182. van Leeuwen CH a., van der Velde G., van Lith B., Klaassen M. 2012. Experimental quantification of long distance dispersal potential of aquatic snails in the gut of migratory birds. PLoS ONE 7.

183. Leite TS., Haimovici M., Mather J. 2009. Octopus insularis (Octopodidae), evidences of a specialized predator and a time-minimizing hunter. Marine Biology 156:2355–2367.

184. Lepre CJ., Roche H., Kent D V., Harmand S., Quinn RL., Brugal J-P., Texier P-J., Lenoble A., Feibel CS. 2011. An earlier origin for the Acheulian. Nature 477:82–85.

185. Lewis DB., Magnuson JJ. 1999. Intraspecific gastropod shell strength variation among north temperate lakes. Canadian Journal of Fisheries and Aquatic Sciences 56:1687–1695.

186. Li F., Wu N., Lu H., Zhang J., Wang W., Ma M., Zhang X., Yang X. 2013. Mid-Neolithic Exploitation of Mollusks in the Guanzhong Basin of Northwestern China: Preliminary Results. PLoS ONE 8.

187. Li R-Y., Young HR., Zhan R-B. 2011. Drilling predation on scaphopods and other molluscs from the Upper Cretaceous of Manitoba, Canada. Palaeoworld 20:296–307.

188. Linseele V., Van Neer W., Thys S., Phillipps R., Cappers R., Wendrich W., Holdaway S. 2014. New Archaeozoological Data from the Fayum “Neolithic” with a Critical Assessment of the Evidence for Early Stock Keeping in Egypt. PLoS ONE 9:e108517.

189. Lombardo U., Szabo K., Capriles JM., May JH., Amelung W., Hutterer R., Lehndorff E., Plotzki A., Veit H. 2013. Early and Middle Holocene Hunter-Gatherer Occupations in Western Amazonia: The Hidden Shell Middens. PLoS ONE 8.

190. Lubell D., Barton N. 2011. Gastropods and humans in the late Palaeolithic and Mesolithic of the western Mediterranean basin. Quaternary International 244:1–4.

191. Mackay A., Stewart B a., Chase BM. 2014. Coalescence and fragmentation in the late Pleistocene archaeology of southernmost Africa. Journal of Human Evolution 72:26–51.

192. Mackay A., Welz A. 2008. Engraved ochre from a Middle Stone Age context at Klein Kliphuis in the Western Cape of South Africa. Journal of Archaeological Science 35:1521–1532.

193. Magnesen T., Redmond KJ. 2011. Potential predation rates by the sea stars Asterias rubens and Marthasterias glacialis, on juvenile scallops, Pecten maximus, ready for sea ranching. Aquaculture International 20:189–199.

194. Maher LA., Richter T., Macdonald D., Jones MD., Martin L., Stock JT. 2012. Twenty Thousand-Year-Old huts at a Hunter-Gatherer settlement in Eastern Jordan. PLoS ONE 7:e31447.

195. Malafouris L. 2008. Beads for a Plastic Mind: the “Blind Man”s Stick’ (BMS) Hypothesis and the Active Nature of Material Culture. Cambridge Archaeological Journal 18:401.

196. Marean CW., Bar-Matthews M., Bernatchez J., Fisher E., Goldberg P., Herries AIR., Jacobs Z., Jerardino A., Karkanas P., Minichillo T., Nilssen PJ., Thompson E., Watts I., Williams HM. 2007. Early human use of marine resources and pigment in South Africa during the Middle Pleistocene. Nature 449:905–908.

197. Marean CW. 2009. The origins and significance of coastal resource use in Africa and Western Eurasia. Journal of Human Evolution journal 77:17–40.

198. Marlowe FW. 2007. Hunting and Gathering: The Human Sexual Division of Foraging Labor. Cross-Cultural Research 41:170–195.

199. Marzke MW. 2013. Tool making, hand morphology and fossil hominins. Philosophical Transactions of the Royal Society B: Biological Sciences 368:20120414.

200. Mather JA., O’Dor RK. 1991. Foraging Strategies and Predation Risk Shape the Natural History of Juvenile Octopus vulgaris. Bulletin of Marine Science 49:256–269.

201. McCall GS. 2006. Multivariate perspectives on change and continuity in the Middle Stone Age lithics from Klasies River Mouth, South Africa. Journal of Human Evolution 51:429–439.

202. McCall GS. 2007. Behavioral ecological models of lithic technological change during the later Middle Stone Age of South Africa. Journal of Archaeological Science 34:1738–1751.

203. Meredith-Williams M., Hausmann N., Inglis R., Bailey G. 2014. 4200 New Shell Mound Sites in the Southern Red Sea. Internet Archaeology.

204. Mijares ASB. 2005. The Archaeology of Peñablanca Cave Sites , Northern Luzon, Philippines. Journal of Austronesian Studies 1:65–92.

205. Miller JM., Willoughby PR. 2014. Radiometrically dated ostrich eggshell beads from the Middle and Later Stone Age of Magubike Rockshelter, southern Tanzania. Journal of Human Evolution 74:118–122.

206. Mind M. 1959. Modern Mind. Nature 183:953–953.

207. Mirazón Lahr M. 2016. The shaping of human diversity: filters, boundaries and transitions. Philosophical transactions of the Royal Society of London. Series B, Biological sciences 371:62–108.

208. Morse K. 1993. Shell beads from Mandu Mandu Creek rock-shelter, Cape Range peninsula, Western Australia, dated before 30,000 b.p. Antiquity 67:877–883.

209. Morton B., Peharda M., Harper E. 2007. Drilling and chipping patterns of bivalve prey predation by Hexaplex trunculus (Mollusca: Gastropoda: Muricidae). Journal of the Marine Biological Association of the UK 87:933–940.

210. Mowles SL., Rundle SD., Cotton PA. 2011. Susceptibility to predation affects trait-mediated indirect interactions by reversing interspecific competition. PloS one 6:e23068.

211. Muscarella OW. 2006. The Excavation of Hasanlu: An Archaeological Evaluation. Bulletin of the American Schools of Oriental Research 342:69–94.

212. Nigra BT., Arnold JE. 2013. Explaining the monopoly in shell-bead production on the Channel Islands: Drilling experiments with four lithic raw materials. Journal of Archaeological Science 40:3647–3659.

213. Nowell A. 2010. Defining Behavioral Modernity in the Context of Neandertal and Anatomically Modern Human Populations. Annual Review of Anthropology 39:437–452.

214. O’Connor S., Robertson G., Aplin KP. 2014. Are osseous artefacts a window to perishable material culture?? Implications of an unusually complex bone tool from the Late Pleistocene of East Timor. Journal of Human Evolution 67:108–119.

215. Olaberria J-P. 2014. The Conception of Hull Shape by Shell-builders in the Ancient Mediterranean. International Journal of Nautical Archaeology 43:351–368.

216. Oliva M., Yll R. 2010. The use of marine shell in Cingle Vermell and Roc del Migdia (Vilanova de Sau, Barcelona, Spain), two prehistoric sites in the western Mediterranean. First approach. In: Álvarez-Fernández E, Carcajal-Contreras D eds. Not only food. Marine, Terrestrial and Freshwater Molluscs in Archaeological Sites. Proceedings of the 2nd Meeting of the ICAZ Archaeomalacology Working Group (Santander, February 19th – 22nd 2008). Donostia: Aranzadi Zientzia Elkartea, 138–145.

217. Palmer AR., Strobeck C., Palmer AR., Palmer AR. 2003. FA Analyses Revisited. In: Polak M ed. Developmental Instability (DI): Causes and Consequences. Oxford University Press, 279–319.

218. Parr WCH., Chamoli U., Jones A., Walsh WR., Wroe S. 2013. Finite element micro-modelling of a human ankle bone reveals the importance of the trabecular network to mechanical performance: New methods for the generation and comparison of 3D models. Journal of Biomechanics 46:200–205.

219. Passini G., Garassino A. 2012. Naticid gastropod and octopodid cephalopod predatory traces: evidence of drill holes on the leucosid crab Ristoria pliocaenica (Ristori, 1891), from the Pliocene of the “La Serra” quarry (Tuscany, Italy). Atti della Società italiana di scienze naturali e del Museo civico di storia naturale di Milano 153:257–266.

220. Peacock E., Randklev CR., Wolverton S., Palmer R., Zaleski S. 2012. The “cultural filter,” human transport of mussel shell, and the applied potential of zooarchaeological data. Ecological Applications 22:1446–1459.

221. Peacock E., Haag WR., Warren ML. 2005. Prehistoric decline in freshwater mussels coincident with the advent of maize agriculture. Conservation Biology 19:547–551.

222. Pechenik JA., Lewis S. 2000. Avoidance of drilled gastropod shells by the hermit crab Pagurus longicarpus at Nahant, Massachusetts. Journal of Experimental Marine Biology and Ecology 253:17–32.

223. Perera N., Kourampas N., Simpson I a., Deraniyagala SU., Bulbeck D., Kamminga J., Perera J., Fuller DQ., Szabó K., Oliveira N V. 2011. People of the ancient rainforest: Late Pleistocene foragers at the Batadomba-lena rockshelter, Sri Lanka. Journal of Human Evolution 61:254–269.

224. Perz R., Toczyski J., Kindig M., Ito D., Ejima S., Kamiji K., Yasuki T., Crandall J., Subit D. 2013. Evaluation of the Geometrical Properties Distribution Along the Human Ribs Using Different X‐Ray Imaging Methods. In: IRCOBI Conference 2013. 245–256.

225. von Petzinger G., Nowell A. 2014. A place in time: Situating Chauvet within the long chronology ofsymbolic behavioral development. Journal of Human Evolution 74:37–54.

226. Pietak LM. 1998. Body Symbolism and Cultural Aesthetics: The use of Shell Beads and Ornaments by Delaware and Munsee Groups. North American Archaeologist 19:135–161.

227. Plug I. 1982. Bone Tools and Shell, Bone and Ostrich Eggshell Beads from Bushman Rock Shelter (BRS), Eastern Transvaal. The South African Archaeological Bulletin 37:57.

228. Poole K. 2015. Foxes and Badgers in Anglo-Saxon Life and Landscape. Archaeological Journal 172:389–422.

229. Porraz G., Parkington JE., Rigaud JP., Miller CE., Poggenpoel C., Tribolo C., Archer W., Cartwright CR., Charrié-Duhaut A., Dayet L., Igreja M., Mercier N., Schmidt P., Verna C., Texier PJ. 2013. The MSA sequence of Diepkloof and the history of southern African Late Pleistocene populations. Journal of Archaeological Science 40:3542–3552.

230. Powell A., Shennan S., Thomas MG. 2009. Late Pleistocene Demography and the Appearance of Modern Human Behavior. Science 324.

231. Quensen J., Woodruff D. 1997. Associations Between Shell Morphology and Land Crab Predation in the Land Snail Cerion. Functional Ecology 11:464–471.

232. Ramsay K., Richardson C., Kaiser M. 2001. Causes of shell scarring in dog cockles Glycymeris glycymeris L. Journal of Sea Research 45:131–139.

233. Reese DS. 1991. the Trade of Indo-Pacific Shells Into the Mediterranean Basin and Europe. Oxford Journal of Archaeology 10:159–196.

234. Reimchen TE. 1982. Shell size divergence in Littorina mariae and L . obtusata and predation by crabs. Canadian Journal of Zoology 60:687–695.

235. Reynard JP. 2013. Trampling in coastal sites: An experimental study on the effects of shell on bone in coastal sediment. Quaternary International 330:156–170.

236. Reynolds PD. 2002. Molluscan Radiation - Lesser-known Branches. In: Advances in Marine Biology. Advances in Marine Biology. Amsterdam: Academic Press, 137–236.

237. Rick TC., Vellanoweth RL., Erlandson JM. 2005. Radiocarbon dating and the “old shell” problem: Direct dating of artifacts and cultural chronologies in coastal and other aquatic regions. Journal of Archaeological Science 32:1641–1648.

238. Rigaud S., d’Errico F., Vanhaeren M., Neumann C. 2009. Critical reassessment of putative Acheulean Porosphaera globularis beads. Journal of Archaeological Science 36:25–34.

239. Rigaud S., d’Errico F., Vanhaeren M., Peñalber X. 2014. A short-term, task-specific site: Epipalaeolithic settlement patterns inferred from marine shells found at Praileaitz I (Basque Country, Spain). Journal of Archaeological Science 41:666–678.

240. Rogalla NS., Amler MRW. 2007. Statistic approach on taphonomic phenomena in shells ofGlycymeris glycymeris (Bivalvia: Glycymerididae) and its significance in the fossil record. Paläontologische Zeitschrift 81:334–355.

241. Rosin ZM., Olborska P., Surmacki A., Tryjanowski P. 2011. Differences in predatory pressure on terrestrial snails by birds and mammals. Journal of Biosciences 36:691–699.

242. Rosin ZM. 2013. Różnice w behawiorze i cechach muszli form barwnych wstężyka gajowego Cepaea nemoralis ( L .) a preferencje pokarmowe ptaków i drobnych ssaków Zuzanna Maria Rosin Rozprawa doktorska. Adam Mickiewicz University in Poznań.

243. Rosin ZM., Kobak J., Lesicki A., Tryjanowski P. 2013. Differential shell strength of Cepaea nemoralis colour morphs - Implications for their anti-predator defence. Naturwissenschaften 100:843–851.

244. Rossano MJ. 2010. Making Friends, Making Tools, and Making Symbols. Current Anthropology 51:S89–S98.

245. Rybin EP. 2014. Tools, beads, and migrations: Specific cultural traits in the Initial Upper Paleolithic of Southern Siberia and Central Asia. Quaternary International 347:39–52.

246. Sawyer JA., Zuschin M., Riedel B., Stachowitsch M. 2009. Predator-prey interactions from in situ time-lapse observations of a sublittoral mussel bed in the Gulf of Trieste (Northern Adriatic). Journal of Experimental Marine Biology and Ecology 371:10–19.

247. Sawyer JA. 2010. Quantitative studies of drilling predation on Cenozoic and Recent marine molluscs from Europe.

248. Serrand N., Vigne J., Guilaine J. 2005. Early Preceramic Neolithic marine shells from Shillourokambos, Cyprus (late 9th-8th mill. cal BC): a mainly-ornamental set with similarities to mainland PPNB. In: Bar-Yosef-Mayer D ed. Archaeomalacology. Molluscs in former environments of human behaviour. Oxford: Oxbow Books, 122–129.

249. Silva ACF., Hawkins SJ., Boaventura DM., Thompson RC. 2008. Predation by small mobile aquatic predators regulates populations of the intertidal limpet Patella vulgata (L.). Journal of Experimental Marine Biology and Ecology 367:259–265.

250. Sinitsyn A. 2003. A Palaeolithic “Pompeii” at Kostenki, Russia. Antiquity 77:9–14.

251. Sládek V., Hora M., Farkašová K., Rocek TR. 2016. Impact of grinding technology on bilateral asymmetry in muscle activity of the upper limb. Journal of Archaeological Science 72:142–156.

252. Slice D. 2005. Modern morphometrics in physical anthropology. Chicago: Kluwer Academic.

253. Smith CD. 2003. Diet of Octopus vulgaris in False Bay, South Africa. Marine Biology 143:1127–1133.

254. Solana DC., Zugasti IG. 2011. The use of mollusc shells as tools by coastal human groups: The contribution of ethnographical studies to research on mesolithic and early neolithic technologies in Northern Spain. Journal of Anthropological Research 67:77–102.

255. Stafford ES., Leighton LR. 2011. Vermeij Crushing Analysis: A new old technique for estimating crushing predation in gastropod assemblages. Palaeogeography, Palaeoclimatology, Palaeoecology 305:123–137.

256. Sterelny K. 2014. A Paleolithic Reciprocation Crisis: Symbols, Signals, and Norms. Biological Theory 9:65–77.

257. Stiner M. 2010. Shell ornaments from the Upper Paleolithic and Mesolithic layers of Klissoura Cave 1 by Prosymnia, Greece. Eurasian Prehistory 7:287–308.

258. Stiner MC. 2013. An Unshakable Middle Paleolithic? Current Anthropology 54:S288–S304.

259. Stiner MC. 2014. Finding a Common Bandwidth: Causes of Convergence and Diversity in Paleolithic Beads. Biological Theory 9:51–64.

260. Stiner MC., Kuhn SL. 2009. Paleolithic Diet and the Division of Labor in Mediterranean Eurasia. In: Hublin J-J, Richards MP eds. The Evolution of Hominid Diets: Integrating Approaches to the Study of Palaeolithic Subsistence; J.-J. Hublin and M.P. Richards (eds.). Springer Science + Business Media, 155–168.

261. Stiner MC., Kuhn SL., Güleç E. 2013. Early Upper Paleolithic shell beads at Uçaĝizli Cave I (Turkey): Technology and the socioeconomic context of ornament life-histories. Journal of Human Evolution 64:380–398.

262. Stiner MC., Munro ND. 2011. On the evolution of diet and landscape during the Upper Paleolithic through Mesolithic at Franchthi Cave (Peloponnese, Greece). Journal of Human Evolution 60:618–636.

263. Stringer C., Barton RNE. 2008. Putting North Africa on the Map of Modern Human Origins. Evolutionary Anthropology 17:5–7.

264. Szabo K. 2004. Technique and Practice. Shell-working in the Western Pacific and Island Southeast Asia. Research School of Pacific and Asian Studies.

265. Szabo K., Brumm A., Bellwood P. 2007. Shell Artefact Production at 32,000–28,000 BP in Island Southeast Asia: Thinking across Media? Current Anthropology 48:701–723.

266. Taraschewski H., Paperna I. 1982. Trematode infections in Pirenella conica in three sites of a mangrove lagoon in Sinai. Zeitschrift für Parasitenkunde (Berlin, Germany) 67:165–173.

267. Tátá F., Cascalheira J., Marreiros J., Pereira T., Bicho N. 2014. Shell bead production in the Upper Paleolithic of Vale Boi (SW Portugal): An experimental perspective. Journal of Archaeological Science 42:29–41.

268. Tattersall I., Osteology I. 2009. Human origins: Out of Africa. Proceedings of the National Academy of Sciences of the United States of America 106:16018–16021.

269. Texier P-J., Porraz G., Parkington J., Rigaud J-P., Poggenpoel C., Miller C., Tribolo C., Cartwright C., Coudenneau A., Klein R., Steele T., Verna C. 2010. From the Cover: A Howiesons Poort tradition of engraving ostrich eggshell containers dated to 60,000 years ago at Diepkloof Rock Shelter, South Africa. Proceedings of the National Academy of Sciences of the United States of America 107:6180–6185.

270. Texier PJ., Porraz G., Parkington J., Rigaud JP., Poggenpoel C., Tribolo C. 2013. The context, form and significance of the MSA engraved ostrich eggshell collection from Diepkloof Rock Shelter, Western Cape, South Africa. Journal of Archaeological Science 40:3412–3431.

271. Thakar HB. 2011. Intensification of shellfish exploitation: Evidence of species-specific deviation from traditional expectations. Journal of Archaeological Science 38:2596–2605.

272. Thomas KD. 2015. Molluscs emergent , part II: themes and trends in the scientific investigation of molluscs and their shells as past human resources. 44:1–9.

273. Trubitt MBD. 2013. The production and exchange of marine shell prestige goods. Journal of Archaeological Research 11:243–277.

274. Trupp TL. 2007. Looking for the Individual: An Examination of Personal Adornment in the European Upper Palaeolithic. University of Victoria All.

275. Turra A., Denadai MR., Leite FPP. 2005. Predation on gastropods by shell-breaking crabs: Effects on shell availability to hermit crabs. Marine Ecology Progress Series 286:279–291.

276. Tyler CL., Leighton LR., Carlson SJ., Huntley JW., Kowalewski M. 2014. Predation on Modern and Fossil Brachiopods: Assessing Chemical Defenses and Palatability. Palaios 28:724–735.

277. Valley C., Dahdul M. 2002. Beads and Pendants from Southern California. Pacific Coast Archaeological Society Quarterly 38:47–64.

278. Vanhaeren M., d’Errico F., Billy I., Grousset F. 2004. Tracing the source of Upper Palaeolithic shell beads by strontium isotope dating. Journal of Archaeological Science 31:1481–1488.

279. Vanhaeren M., D’Errico F., Stringer C., James SL., Todd JA., Mienis HK. 2006a. Middle Paleolithic shell beads in Israel and Algeria. Science 312:1785–1788.

280. Vanhaeren M., d’Errico F., Stringer CB., James SL., Todd JA., Mienis HK. 2006b. Middle Paleolithic Shell Beads in Israel and Algeria. Science 312:1785–1789.

281. Vanhaeren M., D’Errico F., van Niekerk KL., Henshilwood CS., Erasmus RM. 2013. Thinking strings: Additional evidence for personal ornament use in the Middle Stone Age at Blombos Cave, South Africa. Journal of Human Evolution 64:500–517.

282. Vanhaeren M., d’Errico F. 2005. Grave goods from the Saint-Germain-la-Rivière burial: Evidence for social inequality in the Upper Palaeolithic. Journal of Anthropological Archaeology 24:117–134.

283. Vanhaeren M., D’Errico F. 2011. L’émergence du corps paré. Civilisations 59:59–86.

284. Varela HH., Cocilovo JA., Santoro CM., Rothhammer F. 2006. Microevolution of human archaic groups of Arica, northern Chile, and its genetic contribution to populations from the Formative period. Revista Chilena de Historia Natural 79:185–193.

285. Vellanoweth R. 2001. AMS Radiocarbon Dating and Shell Bead Chronologies : Middle Holocene Trade and Interaction in Western North America. Journal of Archaeological Science 28:941–950.

286. Villa P., Soressi M., Henshilwood CS., Mourre V. 2009. The Still Bay points of Blombos Cave (South Africa). Journal of Archaeological Science 36:441–460.

287. Vita-Finzi C., Stringer C. 2007. The setting of the Mt. Carmel caves reassessed. Quaternary Science Reviews 26:436–440.

288. Van Vlack K. 2010. The Island Chumash: Behavioral Ecology of a Maritime Society. Transforming Anthropology 18:195–196.

289. Vokes AW. 1974. Shell Artifacts. In: Thiel JH, Mabry JB eds. 2006 – Rio Nuevo Archaeology Program, 2000–2003: Investigations at the San Agustín Mission and Mission Gardens, Tucson Presidio, Tucson Pressed Brick Company, and Clearwater Site. Technical Report No. 2004-11. Tucson: Center for Desert Archaeology,.

290. Wada S., Kawakami K., Chiba S. 2012. Snails can survive passage through a bird’s digestive system. Journal of Biogeography 39:69–73.

291. Wadley L. 2005. Putting ochre to the test: Replication studies of adhesives that may have been used for hafting tools in the Middle Stone Age. Journal of Human Evolution 49:587–601.

292. Wadley L. 2007. Announcing a Still Bay industry at Sibudu Cave, South Africa. Journal of Human Evolution 52:681–689.

293. Wadley L. 2010. Cemented ash as a receptacle or work surface for ochre powder production at Sibudu, South Africa, 58,000 years ago. Journal of Archaeological Science 37:2397–2406.

294. Wadley L., Sievers C., Bamford M., Goldberg P., Berna F., Miller C. 2011. Middle Stone Age Bedding Construction and Settlement Patterns at Sibudu, South Africa. Science 334.

295. Wadley L. 2012. Two “moments in time” during Middle Stone Age occupations of Sibudu, South Africa. Southern African Humanities 24:79–97.

296. Wadley L., Mohapi M. 2008. A Segment is not a Monolith: evidence from the Howiesons Poort of Sibudu, South Africa. Journal of Archaeological Science 35:2594–2605.

297. Walker SE., Brett CE. 2002. Post-Paleozoic Patterns in Marine Predation Post-Paleozoic Patterns in Marine Predation: Was There a Mesozoic and Cenozoic Marine Predatory Revolution? Paleontological Society Papers 8:119–194.

298. Watts I. 2010. The pigments from Pinnacle Point Cave 13B, Western Cape, South Africa. Journal of Human Evolution 59:392–411.

299. Wesselingh FP., Cadée GC., Renema W. 1999. Flying high: On the airborne dispersal of aquatic organisms as illustrated by the distribution histories of the gastropod genera Tryonia and Planorbarius. Geologie en Mijnbouw/Netherlands Journal of Geosciences 78:165–174.

300. Wild EM., Neugebauer-Maresch C., Einwögerer T., Stadler P., Steier P., Brock F. 2008. 14C Dating of the Upper Paleolithic Site at Krems-Hundssteig in Lower Austria. Radiocarbon 50:1–10.

301. Wilkens B. 2005. The use of marine shells at Sumhuram, Oman. In: Bar-Yosef-Mayer D ed. Archaeomalacology. Molluscs in former environments of human behaviour. Oxford: Oxbow Books, 159–165.

302. Wilkins J. 2010. Style, symboling, and interaction in Middle Stone Age societies. vis-à-vis: Explorations in Anthropology 10:102–125.

303. Willey GR. 1945. Horizon Styles and Pottery Traditions in Peruvian Archaeology. American Antiquity 11:49.

304. Wind J. 2005. The evolutionary history of the human speech organs. In: Wind J, Pulleyblank EG, de Grolier E, Bichakjian BH eds. Studies in Language Origins. John Benjamins Publishing Company, 173–198.

305. Wrinn PJ., Rink WJ. 2003. ESR Dating of Tooth Enamel From Aterian Levels at Mugharet el ‘Aliya (Tangier, Morocco). Journal of Archaeological Science 30:123–133.

306. Wynn T. 2009. Hafted spears and the archaeology of mind. Proceedings of the National Academy of Sciences of the United States of America 106:9544–9545.

307. Wynn T., Coolidge FL. 2004. The expert Neandertal mind. Journal of Human Evolution 46:467–487.

308. Zagyvai Á., Demeter G., Zagyvai A., Demeter G. 2008. Tracing prey-predatory interactions in the Early Sarmatian (Mid-Miocene) shely community from Rollsdorf Formation, Waldhof, Austria based on bioerosional observations. Acta GGM Debrenica 3:51–60.

309. Zaidel DW., Nadal M., Flexas A., Munar E. 2013. An Evolutionary Approach to Art and Aesthetic Experience. Psychology of Aesthetics, Creativity, and the Arts 7:100–109.

310. Zhou ZY., Guan Y., Gao X., Wang CX. 2013. Heat treatment and associated early modern human behaviors in the Late Paleolithic at the Shuidonggou site. Chinese Science Bulletin 58:1801–1810.

311. Zilhao J. 2010. Neanderthals are us: genes and culture. Radical Anthropology 4:4–15.

312. Zilhão J. 2007. The emergence of ornaments and art: An archaeological perspective on the origins of “behavioral modernity.” Journal of Archaeological Research 15:1–54.

313. Zilhão J., Angelucci DE., Badal-García E., d’Errico F., Daniel F., Dayet L., Douka K., Higham TFG., Martínez-Sánchez MJ., Montes-Bernárdez R., Murcia-Mascarós S., Pérez-Sirvent C., Roldán-García C., Vanhaeren M., Villaverde V., Wood R., Zapata J. 2010. Symbolic use of marine shells and mineral pigments by Iberian Neandertals. Proceedings of the National Academy of Sciences of the United States of America 107:1023–1028.

314. Zugasti FIG. 2011a. The Use of Echinoids and Crustaceans as Food During the Pleistocene-Holocene Transition in Northern Spain: Methodological Contribution and Dietary Assessment. The Journal of Island and Coastal Archaeology 6:115–133.

315. Zugasti FIG. 2011b. Early Holocene land snail exploitation in northern spain: the case of La Fragua cave. Environmental Archaeology 16:36–48.

316. Zuschin M., Stachowitsch M., Stanton RJ. 2003. Patterns and processes of shell fragmentation in modern and ancient marine environments. Earth-Science Reviews 63:33–82.
